# Assisted Evolution Enables HIV-1 to Overcome a High TRIM5α-Imposed Genetic Barrier to Rhesus Macaque Tropism

**DOI:** 10.1371/journal.ppat.1003667

**Published:** 2013-09-26

**Authors:** Steven J. Soll, Sam J. Wilson, Sebla B. Kutluay, Theodora Hatziioannou, Paul D. Bieniasz

**Affiliations:** 1 Laboratory of Retrovirology, The Rockefeller University, New York, New York, United States of America; 2 Aaron Diamond AIDS Research Center, The Rockefeller University, New York, New York, United States of America; 3 Howard Hughes Medical Institute, The Rockefeller University, New York, New York, United States of America; University of Massachusetts Medical School, United States of America

## Abstract

Diversification of antiretroviral factors during host evolution has erected formidable barriers to cross-species retrovirus transmission. This phenomenon likely protects humans from infection by many modern retroviruses, but it has also impaired the development of primate models of HIV-1 infection. Indeed, rhesus macaques are resistant to HIV-1, in part due to restriction imposed by the TRIM5α protein (rhTRIM5α). Initially, we attempted to derive rhTRIM5α-resistant HIV-1 strains using two strategies. First, HIV-1 was passaged in engineered human cells expressing rhTRIM5α. Second, a library of randomly mutagenized capsid protein (CA) sequences was screened for mutations that reduced rhTRIM5α sensitivity. Both approaches identified several individual mutations in CA that reduced rhTRIM5α sensitivity. However, neither approach yielded mutants that were fully resistant, perhaps because the locations of the mutations suggested that TRIM5α recognizes multiple determinants on the capsid surface. Moreover, even though additive effects of various CA mutations on HIV-1 resistance to rhTRIM5α were observed, combinations that gave full resistance were highly detrimental to fitness. Therefore, we employed an ‘assisted evolution’ approach in which individual CA mutations that reduced rhTRIM5α sensitivity without fitness penalties were randomly assorted in a library of viral clones containing synthetic CA sequences. Subsequent passage of the viral library in rhTRIM5α-expressing cells resulted in the selection of individual viral species that were fully fit and resistant to rhTRIM5α. These viruses encoded combinations of five mutations in CA that conferred complete or near complete resistance to the disruptive effects of rhTRIM5α on incoming viral cores, by abolishing recognition of the viral capsid. Importantly, HIV-1 variants encoding these CA substitutions and SIV_mac239_ Vif replicated efficiently in primary rhesus macaque lymphocytes. These findings demonstrate that rhTRIM5α is difficult to but not impossible to evade, and doing so should facilitate the development of primate models of HIV-1 infection.

## Author Summary

Retroviruses such as HIV-1 often exhibit limited capacity to infect species other than their natural hosts. This phenomenon is partly due to the existence of antiviral proteins that protect against infection by viruses that have not adapted to a particular species. For example, the resistance of rhesus macaques, the monkey species most commonly used in medical research, to HIV-1 infection is partly attributable to the vulnerability of HIV-1 to TRIM5α. Rhesus macaque TRIM5α (rhTRIM5α) blocks HIV-1 infection by recognition of the viral capsid following its entry into the cell, and it has proven difficult to derive HIV-1 strains that are resistant to rhTRIM5α. However, by devising an ‘assisted evolution’ approach, we identified particular combinations of mutations that render HIV-1 resistant to rhTRIM5α. These mutations enable HIV-1 to evade rhTRIM5α by abolishing recognition of the capsid. Notably, introduction of rhTRIM5α-resistant capsids into an HIV-1 that was also engineered to avoid the rhesus macaque APOBEC3 antiviral proteins, allowed efficient HIV-1 replication in rhesus macaque lymphocytes. These discoveries have the potential to advance the development of rhesus macaque models of HIV-1 infection.

## Introduction

The narrow species tropism of HIV-1 is, in part, caused by species-specific variation in restriction factors that inhibit retroviral infection. This fact has important corollaries, one of which is that humans are likely protected from infection by many retroviruses. Conversely, many animal species commonly used in biomedical research cannot be infected by HIV-1, imposing severe limitations on the development of non-human primate models of HIV-1 infection and pathogenesis [1]. One antiretroviral protein that limits HIV-1 tropism is TRIM5α, a restriction factor that was initially identified in a screen of rhesus macaque (rh, *M. mulatta*) genes for inhibitors of HIV-1 infection [2], but in fact can have broad antiretroviral activity [3]–[8]. As a member of a large family of tri-partite motif (TRIM) containing proteins, TRIM5α contains N-terminal RING, B-box, and coiled-coil domains [9]. TRIM5α, like other restriction factors such as tetherin and APOBEC3 proteins, inhibits HIV-1 in a species-specific manner [10]–[17]. In particular, HIV-1 is vulnerable to restriction by rhTRIM5α but is resistant to restriction by human TRIM5α due to sequence differences in their C-terminal PRY/SPRY domains [18]–[23]. TRIM5α functions by targeting the incoming viral capsid within minutes of viral entry into the cell cytoplasm [24]. Viral capsids are likely directly bound by the PRY/SPRY domain during restriction [25]–[28] leading to inactivation of the viral core, followed by dissolution and, in some cases, degradation of viral core components [27], [29], [30].

In several instances, restriction factors target generic viral or cellular features that are required by retroviruses (such as lipid membranes, dNTP pools, and RNA) in a manner that is not directly affected by viral RNA or protein sequence. These nonspecific mechanisms by which restriction factors act makes viral escape difficult, and the major way that retroviruses evade such inhibitors is through the acquisition and adaptation of specific antagonists [31]. For example, the viral proteins Vpu, Nef, Vif and Vpx are able to rescue HIV-1 or SIV from restriction by human tetherin, APOBEC3 and SamHD1 proteins by targeting the restriction factors for removal from their sites of action, or for degradation [10], [11], [13]–[15], [32]–[38]. In contrast, TRIM5α proteins are unusual among the known antiretroviral restriction factors, because they bind to a specific viral protein (CA). While some TRIM5α proteins, particular those from old world monkeys have broad antiretroviral activity, and can inhibit retroviruses with widely divergent CA sequences [3], [6]–[8], the requirement that a specific protein be recognized affords viruses the opportunity to evade restriction by evolving a CA protein that cannot be recognized. Indeed, transplantation of SIV_mac239_ CA in the context of a chimeric HIV-1 confers rhTRIM5α resistance [8], [39]. Similarly, sensitivity or resistance of murine leukemia virus to human TRIM5α can be acquired by mutations in CA [5], [6], [40], [41]. Amino acids in CA that confer TRIM5α sensitivity are generally exposed on the surface of the viral core [42], [43] and are therefore accessible for binding to TRIM5α following viral entry into the cell.

While it should be possible for HIV-1 to evade rhTRIM5α via changes in CA sequence, it has proven difficult to isolate mutants of HIV-1 that have this property. A few studies have described mutations in HIV-1 CA that confer modestly reduced sensitivity to macaque (*M. mulatta or M.fascicularis*) TRIM5α proteins [44], [45]. Recently one study showed that replacing the entire predicted surface of HIV-1 CA with SIV_mac239_ sequences (twenty five amino acid changes) conferred insensitivity to rhTRIM5α, but at the cost of an ∼15-fold reduction in single-cycle replication fitness [46]. As yet, no study has reported the identification of mutants that give full or nearly full resistance to rhTRIM5α while retaining viral fitness. A possible reason for this is that some monkey TRIM5α proteins (including rhTRIM5α) are capable of recognizing retroviral CA proteins (e.g. HIV-1 and MLV) of very different sequence [3]–[8]. The molecular basis for this broad antiretroviral specificity, and consequently the difficulty with which rhTRIM5α is evaded, may be that rhTRIM5α interacts with multiple determinants on the 3-dimensional HIV-1 CA protein structure [46], [47]. It is also noteworthy that HIV-1 CA is the most genetically fragile (intolerant of amino acid substitution) protein for which robustness/fragility has been quantitatively measured [48]. Together, these factors may impose a high barrier to the evolution of fit, rhTRIM5α-resistant HIV-1 strains, an in a broader sense may be why TRIM5α proteins have been selected as antiviral proteins during host evolution. If it were straightforward for retroviruses to acquire resistance to TRIM5α, then little selective advantage would be conferred on hosts that employ TRIM5α as an antiretroviral protein.

An appreciation of how and why it is difficult for HIV-1 to acquire resistance to rhTRIM5α is important for a complete understanding of factors limiting transmission of primate lentiviruses among divergent primate species. Additionally, overcoming restriction by rhTRIM5α is required to enable HIV-1 replication in rhesus macaque cells and, thus, for the generation of an animal model that is based on HIV-1 infection of rhesus macaques [39]. Successful simian tropic (st) HIV-1 infection of pig-tailed macaques (*M. nemestrina*) [49], which express a TRIM5-cyclophilin fusion (TRIM-Cyp) that is unable to restrict HIV-1 [50]–[53], demonstrates that the HIV-1 host-range can include macaques when APOBEC3- and TRIM5-imposed restrictions are absent.

In the work presented here, we employed various strategies to identify a number of point mutations in HIV-1 CA that can confer partial resistance to restriction by rhTRIM5α. By combining assortments of these mutations, in an assisted evolution approach, and selecting for viruses that could replicate well in cells expressing rhTRIM5α, we generated mutant HIV-1 capsids that exhibit near-complete resistance to rhTRIM5α and retain fitness. Mutations that conferred resistance to rhTRIM5α were distributed in several different locations over the exterior surface of the HIV-1 capsid, suggesting that rhTRIM5α recognizes several different determinants on HIV-1 CA. The mutations that caused resistance also abolished the ability of rhTRIM5α to cause disintegration of HIV-1 core components, as well as the ability of HIV-1 to saturate rhTRIM5α, suggesting that they exert their effect by preventing rhTRIM5α binding to CA. Notably, when incorporated into an stHIV-1 construct that encodes SIV_mac239_ Vif, rhTRIM5α-resistant CA sequences enabled efficient stHIV-1 replication in primary rhesus macaque lymphocytes.

## Results

### CA evolution during HIV-1 replication in human T-cell lines expressing rhTRIM5α

**Figure 1:**
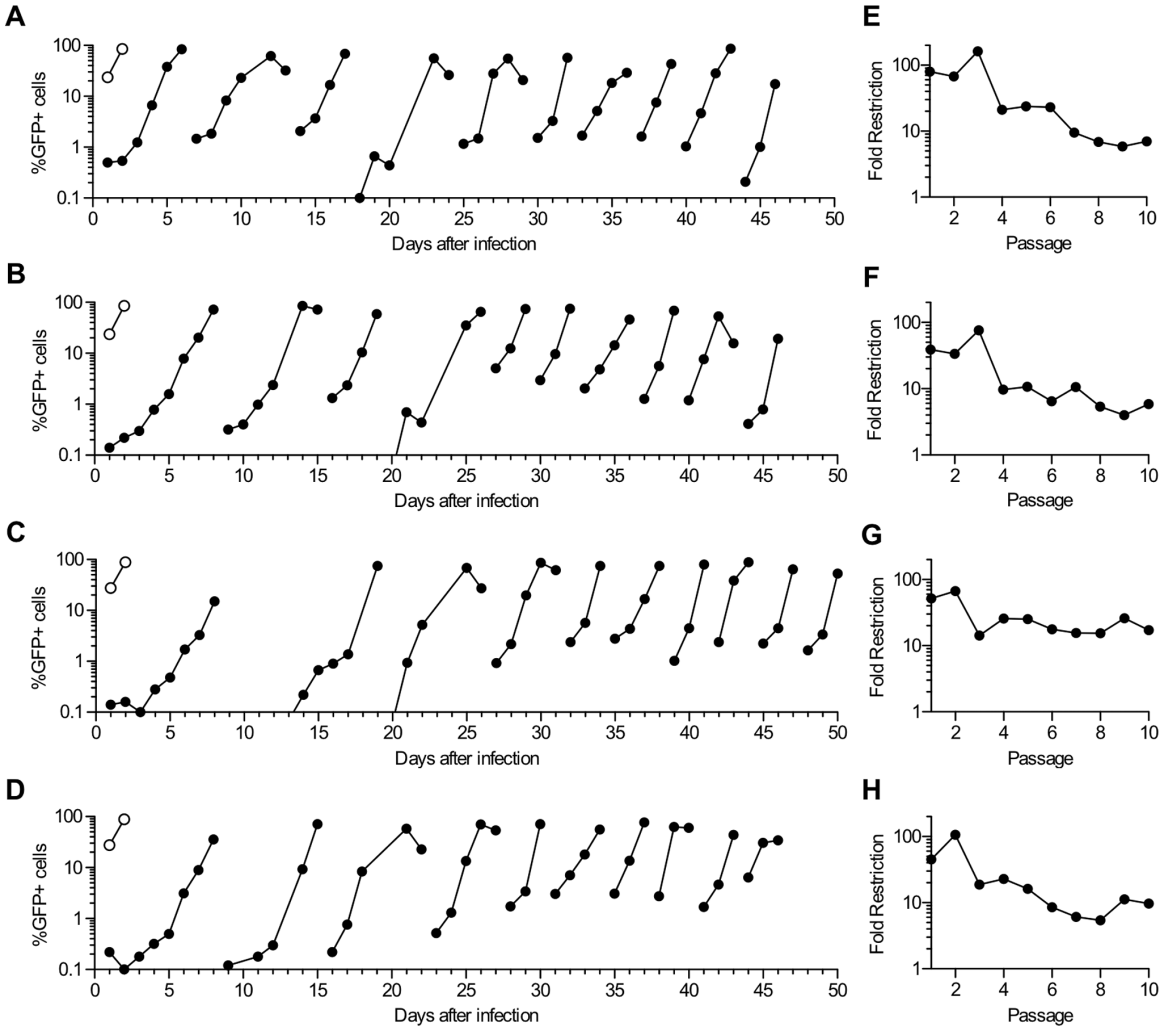
HIV-1 adaptation in human cell lines expressing rhTRIM5α. (A–D) MT2 and MT4 cells expressing either rhTRIM5α (filled symbols) or an empty vector (open symbols) were infected with HIV-1_NL4-3_/HIV-1_HXB2_/GFP (NHG). Aliquots of cells from each culture were withdrawn and fixed for FACS analysis at the indicated times and the percentage of cells that were positive for GFP is plotted versus days after the infection of the first culture. Discontinuities indicate points at which cell-free supernatant was harvested and used to initiate the next passage in TRIM5α-expressing cell lines. NHG was passaged in four different cell lines expressing rhTRIM5α: MT2-rhTRIM5α#8 (A), MT2-rhTRIM5α#15 (B), MT4-rhTRIM5α#29 (C), and MT4-rhTRIM5α#32 (D). (E–H) Viral sensitivity to TRIM5α (Fold restriction) was calculated as the ratio of infectious titers present in culture supernatants at the end of each passage shown on the left, measured on MT2-vector and MT2-rhTRIM5α#8 cells.

To derive HIV-1 CA proteins that confer resistance to rhTRIM5α we first employed an adaptation approach in which HIV-1 was passaged in human cells expressing rhTRIM5α. Initially, a cloned recombinant virus, termed NHG, that contains portions of HIV-1_NL4-3_ and HIV-1_HXB2_, and encodes GFP in place of the Nef gene, was grown in MT2 cells to generate a virus stock with genetic diversity. After fifteen days of replication, cell-free supernatant was used to infect four different clonal cell lines stably expressing rhTRIM5α (two derived from MT2 cells and two derived from MT4 cells) or cells transduced with an empty vector (Figure 1 A–D). The rhTRIM5α variant used belongs to a class of rhTRIM5α variants that encode TFP at amino acids 239–241 and restrict HIV-1 more potently than other variants [54], [55]. The first cycle of HIV-1 replication in these four rhTRIM5α-expressing lines was restricted by 50- to 100-fold relative to that in the empty vector control cells, confirming the potent anti-HIV-1 activity of rhTRIM5α therein (Figure 1 A–D). Infection was monitored by visual inspection of cytopathic effects and by measuring the fraction of GFP-positive cells in each culture at 1 to 3 day intervals. Because MT2 and MT4 cells are exceptionally permissive to HIV-1 replication (they were selected for this experiment for that reason), HIV-1 was able to spread through the four cultures and infect most cells after ∼8 days, despite potent inhibition by rhTRIM5α (Figure 1 A–D). After the majority of cells became GFP-positive and cytopathic effects had become abundant in the cultures, cell free supernatants were used to inoculate fresh rhTRIM5α-expressing cells, and this process was repeated for 10 passages in each of the four cell lines. The time taken for the majority of the cells to become GFP-positive during each passage appeared to decrease as the number of passages increased (Figure 1 A–D), implying adaptation of the virus to the rhTRIM5α expressing cells. At each passage, we measured the infectious HIV-1 titer in the four culture supernatants on both rhTRIM5α expressing and empty vector expressing cell lines. These analyses indicated that there was a progressive decrease in the sensitivity of the virus population to rhTRIM5α as the number of passages on rhTRIM5α expressing cells increased (Figure 1E–H). However, even the passaged virus stock retained at least some degree of sensitivity to rhTRIM5α, and each of the four adapted viruses was restricted by ∼5- to 10-fold after 6 to 8 passages. Sensitivity to rhTRIM5α did not appear to decrease with further passage (Figure 1E–H).

**Figure 2:**
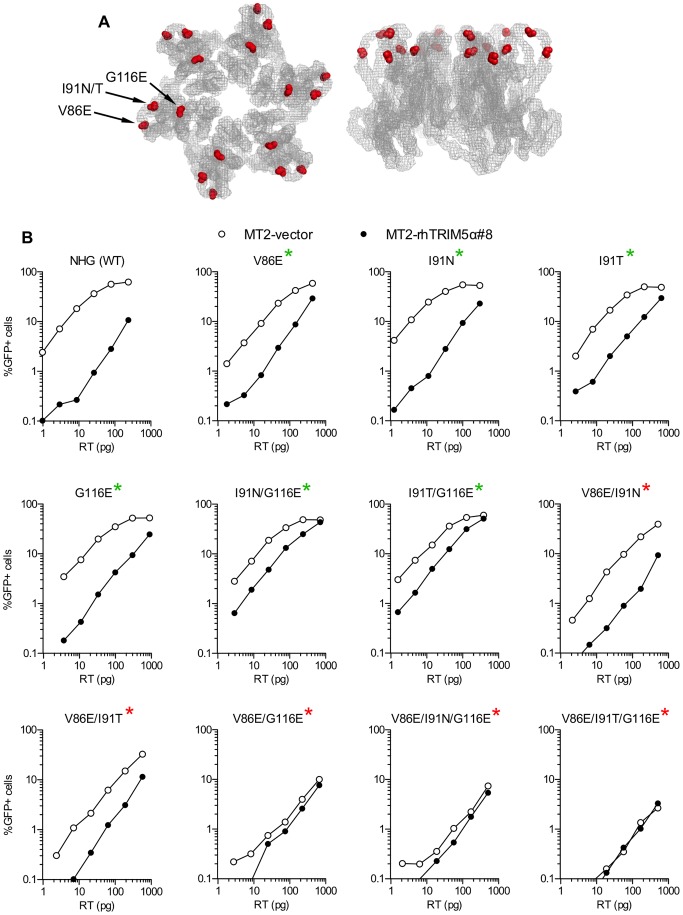
HIV-1 CA mutations selected during passage in rhTRIM5α-expressing cells decrease sensitivity to rhTRIM5α. (A) Positions of amino acid residues at which mutations arose during replication in cells expressing rhTRIM5α are indicated on the HIV-1_NL4-3_ capsid hexameric structure [43]. V86E and I91N/T are in the cyclophilin-binding loop and G116E is in helix six. The capsid hexamer shown on the left represents the exterior of the viral core, viewed from the cytoplasm of an infected cell. The hexamer on the right is oriented with the cytoplasmic face toward the top of the picture and the residues that face toward the interior of the core on the bottom. Images were generated using MacPyMOL. (B) NHG (WT) or NHG derivatives carrying the indicated mutations were titrated on MT2-vector (open symbols) or MT2-rhTRIM5α#8 (filled symbols) cells. Single-cycle infectivity was determined by FACS analysis. Mutant CA sequences that were found in the passaged viral population are marked with a green asterisk while mutants that were not found in the passaged viral population but were constructed using site-directed mutagenesis are marked with a red asterisk.

PCR amplification and bulk sequencing of viral DNA present in the four rhTRIM5α expressing cell cultures after various numbers of passages revealed that four CA mutations (V86E, I91N, I91T, and G116E) were present at a sufficiently high frequency to be detected in bulk sequences (Table 1). These mutations occurred at three different codons, all of which specified amino acids that are exposed on the presumptive outer face of the viral core (Figure 2A). Specifically, amino acids V86 and I91 are both within the cyclophilin-binding loop and G116 is on an outer turn of helix six (Figure 2A). Strikingly, each of the four mutations occurred in each of the four parallel virus cultures. In some cases, the mutant codons were present as mixtures with the WT codons, while in others WT codons became undetectable. In three virus cultures, all four amino acid changes were present in viral populations sampled after ten passages, at which point the WT I91 codon was no longer detected. Conversely, in one viral lineage, mutations at I91 were detected at passage 4, but then disappeared from the culture, and were undetectable after the tenth passage, at which point only V86E and G116E mutations were present as mixtures with the WT codon at both positions. Notably, amino acid substitutions V86M (V86E is described here) and G116E have previously been shown to decrease sensitivity to rhTRIM5α and crab-eating macaque TRIM5α, respectively [44], [45].

**Table 1:** Amino acid changes arising during HIV-1 passage in rhTRIM5α expressing cell lines.

Passage Number	Cell Line			
	MT2#8	MT2#15	MT4#29	MT4#32
P5 (MT2) or P4 (MT4)	V86E I91N,T G116E	V86E I91N G116E	V86E I91N,T G116E	V86E I91N,T G116E
P7 (MT2) or P6 (MT4)	V86E I91N,T G116E	**I91N G116E**	**V86E**	**I91N,T** G116E
P10	V86E **I91N,T** G116E	V86E **I91N,T** **G116E**	V86E G116E	V86E **I91N,T** G116E

Amino acids symbols are **bold** where the WT codon was not detected in the sequence chromatogram. In other cases, the mutant was present as a mixture with the WT codon.

To determine whether and how mutations that arose in CA during passage decreased sensitivity to rhTRIM5α, PCR products amplified from viral cultures were cloned and inserted into the parental pNHG proviral plasmid. The resulting clones included four single CA mutants; V86E, I91N, I91T and G116E, as well as double-mutants I91N/G116E and I91T/G116E. Certain combinations of mutations were not recovered as individual species from the four viral cultures, and so site-directed mutagenesis was used to construct all other possible double and triple mutant combinations of V86E, I91T, I91N and G116E (see below). To test the resulting viruses for sensitivity to rhTRIM5α, single-cycle infection assays were employed, using the MT2 cell lines expressing either rhTRIM5α or an empty vector as targets (Figure 2B).

All four of the single amino acid mutants (V86E, I91N, I91T and G116E) exhibited decreased rhTRIM5α sensitivity. In particular, substitutions V86E and I91T each reduced rhTRIM5α sensitivity from ∼60-fold inhibition to ∼15-fold (Figure 2B). The I91N/G116E and I91T/G116E double mutants exhibited even lower rhTRIM5α sensitivity and were inhibited by less than 10-fold. Notably, the aforementioned mutants all occurred in individual species that were present in the viral culture experiment and exhibited little or no decrease in infectivity on the vector control cell line, suggesting that these individual and combined mutations did not incur major fitness costs. The data obtained with these reconstructed viral clones correlated quite well with the results obtained with each uncloned viral stock harvested after 10 passages in MT2-rhTRIM5α (Figure '1E–H). Specifically, virus populations harvested from MT4-rhTRIM5α#29 carried V86E and G116E, but not I91T or I91N, and this stock was restricted by 16-fold by rhTRIM5α. Conversely the other three uncloned, adapted viruses all carried I91T, I91N, and G116E and were restricted by only 5–10 fold.

We next reconstructed the viral mutants that contained combinations of the aforementioned substitutions that were not detected as individual species in the passage experiment, namely the V86E/I91N, V86E/I91T and V86E/G116E, double mutants and the V86E/I91N/G116E and V86E/I91T/G116E triple mutants. Strikingly, some of these mutants appeared completely, or nearly completely resistant to rhTRIM5α restriction in MT2 cells (Figure 2B). However, those mutants also exhibited severely reduced titers on the control vector expressing MT2 cell line, indicating that large fitness costs were imposed by combinations of mutations that gave rhTRIM5α resistance. The V86E/I91N and V86E/I91T double mutants also had apparent fitness defects that accompanied improvements in resistance to rhTRIM5α. These fitness costs were also observed in the context of spreading replication assays (Figure S1). Specifically, while the partially rhTRIM5α-resistant I91N/G116E and I91T/G116E double mutants were able to replicate as rapidly as WT on control vector expressing MT2 cells, the other combinations of mutations, particularly the V86E/I91N/G116E and V86E/I91T/G116E triple mutants, resulted in attenuated replication (Figure S1). Surprisingly, replication of these triple mutants was inhibited by rhTRIM5α during spreading replication assays (Figure S1), even though they appeared nearly completely resistant in single-cycle infection assays (Figure 2), suggesting the possibility that poor fitness might amplify the effect of residual restriction by rhTRIM5α during spreading replication assays. Notably, the attenuated double and triple mutants exhibited low titer in hamster pgsA cells, but were not sensitive to restriction by human TRIM5α expressed therein (Figure S2). This finding indicates that the apparent fitness defects observed in human cell lines were not due to acquisition of sensitivity to endogenous human TRIM5α, but instead were TRIM5α-independent deficits that coincided with decreases in sensitivity to rhTRIM5α. It therefore appeared that, in spite of the potential for HIV-1 CA to acquire near complete rhTRIM5α resistance via two or three amino acid substitutions, deleterious effects on fitness prevented some combinations of mutations from emerging as individual species during adaptation on rhTRIM5α expressing cell lines.

All four of the capsid amino acid changes that arose during evolution in the rhTRIM5α cell lines were the result of single nucleotide substitutions. We considered the possibility that other amino acid substitutions at the same positions in CA would have conferred a greater degree of rhTRIM5α resistance, but simply did not arise because of their requirement for two or three nucleotide changes in a single codon. To address this possibility, we conducted a vertical mutagenesis experiment, in which we made every possible amino acid substitution at positions 86, 91, and 116 in CA. Thereafter, we screened each of the 57 single amino acid substitutions for rhTRIM5α sensitivity (Figure S3A, B, C). A variety of mutations at positions 86, 91, and 116 decreased TRIM5α sensitivity. For example, at position 91 there were six different amino acid substitutions (I91A, I91P, I91Q, I91Y, I91D, and I91E) that were phenotypically approximately equivalent to I91N and I91T. There were also several amino acid changes at position 91 that conferred a greater degree of TRIM5α resistance than I91N or I91T, but all of them caused decreases in viral titer. For example, the I91G substitution gave near complete rhTRIM5α resistance, but this mutation caused a 70-fold loss in viral titer on empty vector expressing cells (Figure S3B). Several different substitutions at positions 86 and 116 also conferred partial resistance to rhTRIM5α, but often these effects were accompanied by reduced viral titer. At position 86, most substitutions caused reduced rhTRIM5α sensitivity and a glutamine substitution caused the largest increase in viral titer on MT2- rhTRIM5α (Figure S3A). However, when V86Q was combined with substitutions at I91 and/or G116 it did not exhibit higher viral titers on MT2- rhTRIM5α cells than the double mutants that emerged during the viral evolution experiments described in Figures 1 and 2 (unpublished observations). At position 116, none of the mutants exhibited higher titer on MT2-rhTRIM5α than did G116E (Figure S3C). Thus it appeared that the adaptation experiment selected near-optimal amino acids at positions 86, 91 and 116, but was not capable of selecting CA mutants with combinations of mutations that conferred the desired property.

Because our selection experiments were done in human cells, it was possible that endogenous human TRIM5α could have limited the spectrum of rhTRIM5α-resistance mutations that arose at these positions. Indeed, mutations at positions 86, 91, and 116 have been associated with sensitivity to human TRIM5α in the context of cytotoxic T lymphocyte escape [56]. Therefore, we screened the vertical mutant collection for sensitivity to human TRIM5α (Figure S4 A, B, C). Although some sensitivity to human TRIM5α was observed in some mutants, these effects were minor: while N-MLV was restricted by 160-fold by huTRIM5α, the degree to which some HIV-1 CA mutants were restricted was usually not more than 2- to 3-fold. One mutant, V86W, was restricted 6-fold by huTRIM5α. Therefore, it seems unlikely that endogenous TRIM5α would have profoundly limited evolution at these three positions during the adaptation experiment.

### A screen of a randomly mutated CA library for substitutions conferring rhTRIM5α resistance

**Figure 3:**
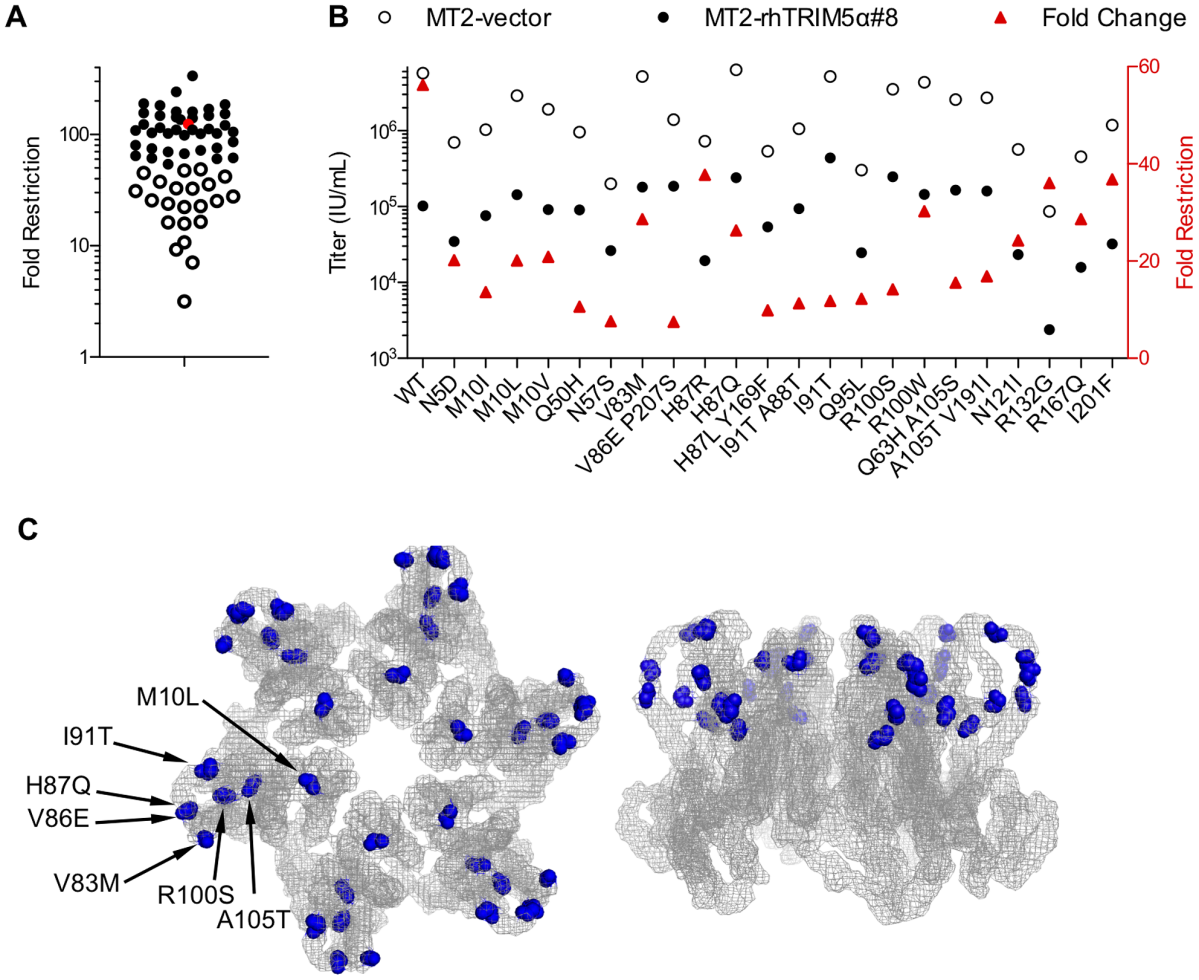
Screen of random HIV-1 CA mutants for rhTRIM5α resistance. (A) NHG clones containing CA substitutions generated by random mutagenesis were used to challenge MT2 cells expressing an empty vector or rhTRIM5α using a single dose of each virus. Fold-restriction by rhTRIM5α (the ratio of the numbers of infected cells in each cell line) is plotted. Only data from viruses that gave measurable infectivity (>0.1% infected cells) in MT2-rhTRIM5α cells are plotted. The WT NHG is plotted as a red symbol and viruses restricted by less than 50-fold are represented by open symbols. (B) NHG mutants carrying CA mutations that decreased TRIM5α sensitivity were retested and infectious titers in MT2-vector (open symbols) and MT2-rhTRIM5α#8 (filled symbols, referencing the left Y-axis) cells, as well as the ratio of these titers (fold restriction red triangles, referencing the right Y-axis) are plotted. (C) Two views of the NL4-3 capsid hexamer [43] are shown, as in Figure 2B. The sites of amino acid substitutions found in (B) to increase infectious titer on MT2-rhTRIM5α cells (M10L, V83M, V86E, H87Q, I91T, R100S and A105T) regardless of effects on titer in vector control cells are indicated.

In a second independent strategy to identify mutations in HIV-1 CA that might confer rhTRIM5α resistance, we screened 91 infectious mutants from a library of NHG clones encoding PCR-mutagenized CA proteins (see Materials and Methods). MT2 cells expressing either the empty vector or rhTRIM5α were infected in a single cycle assay with a single dose of each clonal mutant virus and the ‘fold restriction’ by rhTRIM5α was calculated for those CA mutants that yielded measurable infectivity (>0.1% infected cells) on MT2-rhTRIM5α cells (Figure 3A). The parental virus, NHG was restricted by over 100-fold, as expected, while 22 CA mutant clones were restricted by less than 50-fold. The infectious titer of these 22 mutants was measured on vector expressing and rhTRIM5α expressing cells to confirm their decreased rhTRIM5α sensitivity (Figure 3B). Although there were a variety of mutations that decreased apparent rhTRIM5α sensitivity, many also decreased viral titer on vector-expressing cells. For example, N57S was restricted by only 8-fold by rhTRIM5α, but its infectious titer on vector control cells was reduced 30-fold as compared to the parental NHG virus. Therefore, we selected only mutants that conferred increased titer on rhTRIM5α-expressing cells as candidates that had the potential to contribute to a CA that was both fit and resistant to rhTRIM5α. The infectivity of these mutants on control vector expressing cells was equivalent to or only marginally decreased compared to the parental virus.

Interestingly, two such mutations, V86E and I91T, had also emerged in the viral evolution experiments presented in Figures 1 and 2. Additionally, we selected M10L, M10V, V83M, H87Q, R100S, and A105T as candidate mutations for further analysis because they also caused an increase in infectivity on MT2-rhTRIM5α cells. Of note, each of these amino acid substitutions occurred at positions that are predicted to be exposed to the cytoplasm after viral entry (Figure 3C).

### Fit, rhTRIM5α-resistant viruses, evolved from an assorted pool of CA mutants

While the above experiments demonstrated that a variety of mutations could confer partial resistance to rhTRIM5α, none of the aforementioned single amino acid CA mutants generated fully resistant viruses that did not incur a large fitness cost. However, the I91N/G116E and I91T/G116E double mutants demonstrated the potential for the additive effect of multiple mutations in the acquisition of TRIM5α resistance, without fitness penalties (Figure 2). Conversely, for other double mutants, particularly the combination of V86E and G116E, mutations that conferred partial rhTRIM5α resistance and had little fitness cost on their own, combined to generate rhTRIM5α resistant viruses at the expense of large fitness costs (Figure 2). Thus, it was possible that combinations of mutations that we had identified might generate fully rhTRIM5α-resistant viruses, but it was unpredictable as to whether and which combinations of mutations would allow the maintenance of high viral fitness.

**Figure 4:**
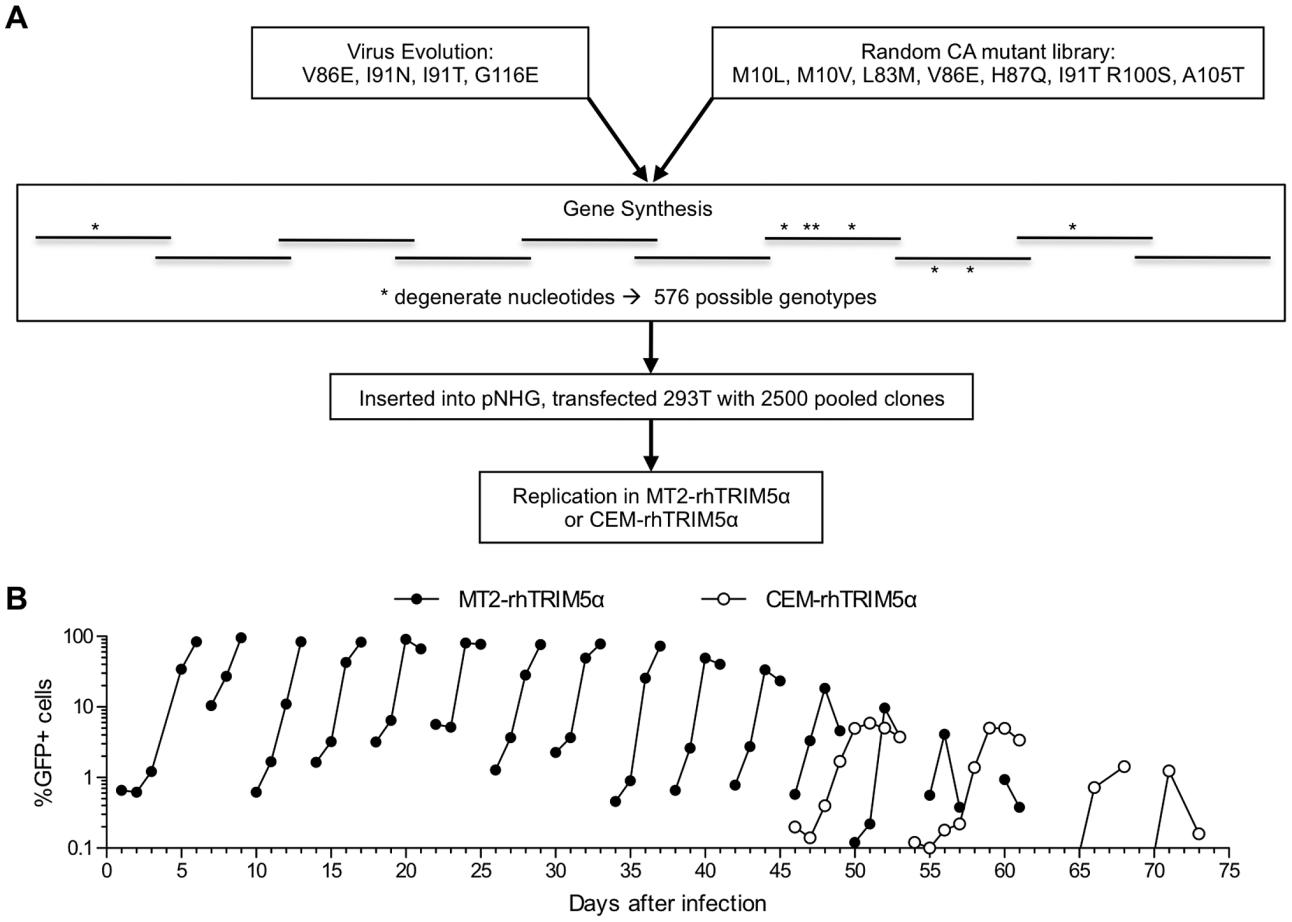
An assisted evolution strategy to derive fit, rhTRIM5α-resistant HIV-1 strains. (A) Schematic representation of the experimental design used to select fit, rhTRIM5α resistant viruses. CA mutations shown to decrease TRIM5α-mediated restriction, without major fitness penalties were incorporated into a synthetic CA amplicon to generate a virus population containing random assortments of the mutations. (B) Serial passages of the virus population described in (A) in MT2-rhTRIM5α cells (filled symbols). Infection was monitored by measuring the proportion of cells expressing GFP over time, as described in the legend to Figure 1. In parallel, the last four of fifteen serial passages were also carried out in CEM-rhTRIM5α cells (open symbols).

Therefore, in an attempt to derive combination mutant CA proteins that were both fully fit and rhTRIM5α resistant, we adopted an ‘assisted evolution’ approach and generated a viral population that contained all possible combinations of the aforementioned non-deleterious mutations that conferred partial TRIM5α resistance (Figure 4A). Overlapping oligonucleotides that contained degenerate nucleotides encoding mixtures of WT and mutant amino acids were used to assemble a synthetic CA library pool containing random assortments of the mutations M10L/V, L83M, V86E, H87Q, I91N/T, R100S, A105T, and G116E. The synthetic CA assortment was used to generate a proviral plasmid library whose theoretical complexity was 576. Proviral plasmid DNA was isolated from 2.5×10^3^ pooled bacterial colonies and transfected as a mixture, yielding a viral population that should contain every possible combination of the above mutations.

This viral population was evolved in MT2-rhTRIM5α through a series of 15 passages (Figure 4B). Additionally, the final four passages were also carried out in CEM-rhTRIM5α cells. The rationale for this was that CEM cells are less permissive than MT2, and we reasoned that this might impose a more stringent requirement for rhTRIM5α resistance and high replicative fitness on the viral population. Unfortunately, beginning at about passage 10, the maximum number of GFP-positive cells began to decrease with each subsequent passage (Figure 4B), presumably as a result of inactivating mutations in the GFP reporter gene in NHG. Nonetheless, visual inspection of cytopathic effects suggested that virus replication accelerated during the serial passage experiment.

We conducted PCR amplification and bulk sequencing of viral DNA from infected cells at passage 4, 9, 11 and 15 (Table 2). This analysis revealed that some mutations were purged from the viral population at varying rates while others apparently became fixed. Additionally, new mutations arose during passage. Specifically, V86E, R100S, and A105T were rapidly purged (by passage 4) while L83M and H87Q were detected at passage 4, 9,and 11, but lost by passage 15. Conversely, mutations M10L, I91N, and G116E became dominant in the viral population, with I91N and G116E rising to dominance rapidly and M10L becoming dominant in MT2 cells (but not CEM cells) between passages 11 and 15. Two additional mutations, namely A92E and M96I, that were not deliberately included in the starting assortment, arose spontaneously and became dominant in the viral population in MT2-rhTRIM5α cells. Curiously, these cyclophilin-binding loop mutations arose at about the same time as two other cyclophilin-binding loop mutations (L83M and H87Q) were lost from the population, suggesting the possibility that these mutations were not compatible with each other. It is also possible that A92E and M96I have the same biological effect as L83M or H87Q but at a lower fitness cost. The apparent lack of heterogeneity at the aforementioned positions in the bulk PCR product suggested that a single CA species containing five mutations (M10L, I91N, A92E, M96I and G116E) had come to dominate the population in MT2-rhTRIM5α cells (Table 2). In CEM-rhTRIM5α cells, the results were slightly different in that a single species did not dominate the population at passage 15. Rather M10L and M96I were present as mixtures with the WT codons, and a third spontaneous mutation, L52I, was identified, again as a mixture with the WT codon.

**Table 2:** Amino acid changes present during passage of HIV-1 encoding an assortment of CA mutations in a rhTRIM5α expressing cell line.

Passage Number	Amino Acid residue:										
	M10	L83	V86	H87	I91	R100	A105	G116	A92	M96	L52
P4	L	M		Q	**N, T**			**E**			
P9	L	M		Q	**N**			**E**	E		
P11	L	M		Q	**N**			**E**	E	I	
P15 (MT2)	**L**				**N**			**E**	**E**	**I**	
P15 (CEM)	L				**N**			**E**	**E**	I	I

Amino acids symbols are **bold** where the WT codon was not detected in the sequence chromatogram. In other cases, the indicated mutation was present as a mixture with the WT codon.

Changes that arose during replication and were not present in the starting assortment (A92E, M96I, L52I) are shown on the right.

**Figure 5:**
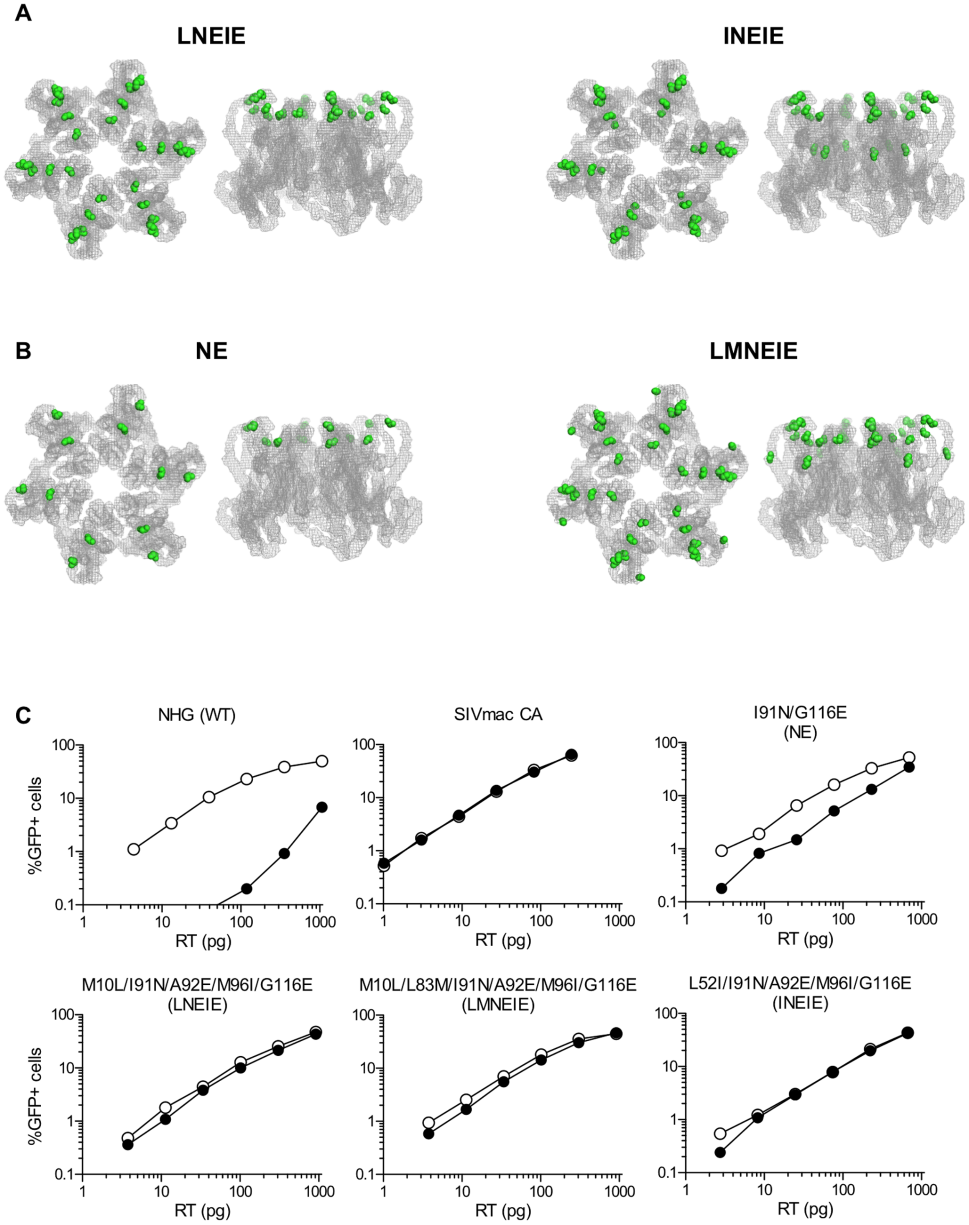
Selection of fit, rhTRIM5α-resistant viral clones from a synthetic CA library containing assortments of mutations conferring partial rhTRIM5α resistance. (A) Two views of the HIV-1 capsid hexamer [43] are shown, as in Figure 2B. The sites of amino acid substitutions in the species (LNEIE) that became dominant in MT2-rhTRIM5α cells, or codominant in CEM-rhTRIM5α cells (LNEIE and INEIE) following the assisted evolution experiment described in Figure 5 are shown. (B) For comparison, the sites of amino acid substitutions in the best performing mutant (NE) derived in the standard selection experiment described in Figure 1 as well as an LNEIE derivative containing an additional substitution (LMNEIE) are also shown. (C) NHG, an NHG derivative encoding an SIV_mac239_ capsid, or NHG derivatives carrying the indicated combination of amino acid substitutions were used to infect MT2-rhTRIM5α#8 cells (filled symbols) or MT2 cells transduced with empty vector (open symbols).

Sequences encoding CA were cloned from the viral DNA present in both MT2-TRIM5α and CEM-TRIM5α cell lines at passage 15. One clone, containing the substitutions M10L/I91N/A92E/M96I/G116E (LNEIE), corresponded to the dominant species in MT2-rhTRIM5α cells and the other, L52I/I91N/A92E/M96I/G116E (INEIE), corresponded to a species that was co-dominant with LNEIE in CEM-rhTRIM5α cells. With the exception of L52I, all of the aforementioned mutations that became fixed, or arose during the passage experiment, were at residues that are predicted to be exposed on the surface of the HIV-1 capsid (Figure 5A).

We next compared the properties of the species that arose via assisted evolution from the assorted CA mutant pool (LNEIE and INEIE, Figure 5A), with the best performing virus species (in terms of fitness and rhTRIM5α resistance) that arose spontaneously during the initial passage in rhTRIM5α expressing cell lines, namely I91N/G116E (NE) (Figure 5B). Additionally, because L83M was among the best performing mutations in the random mutagenesis screen (Figure 3B) and may not have had the opportunity to coexist with the late appearing and proximal A92E and M96I mutations, we also used site-directed mutagenesis to introduce L83M into LNEIE to generate a virus carrying six mutations M10L/L83M/I91N/A92E/M96I/G116E (LMNEIE, Figure 5B).

While the I91N/G116E (NE) double-mutant was restricted in MT2-rhTRIM5α by ∼5-fold, LNEIE, INEIE and LMNEIE were completely or nearly completely resistant to inhibition by rhTRIM5α in single cycle infection assays, similar to a virus expressing the SIV_mac239_ CA (Figure 5C). Moreover in spreading replication assays, LNEIE, LMNEIE, and INEIE replicated with equivalent kinetics in rhTRIM5α-expressing MT2 cells and empty vector expressing MT2 cells (Figure S5). Moreover, the titers and replication kinetics of these viruses on non-restricting MT2 cells were indistinguishable from that of NHG (Figures 5C and S5). Thus, an assisted evolution approach, in which mutations that individually had modest effects on rhTRIM5α were randomly combined and subjected to selection in cell culture, enabled the acquisition of near complete rhTRIM5α resistance, with apparent retention of viral fitness in this cell line.

In some human cells lines, the A92E mutation, which was present in the LNEIE, LMNEIE, and INEIE mutants, has been shown to cause HIV-1 infection to become inhibited by cyclophilin A (CypA). Consequently, infection by A92E CA mutant viruses is increased in the presence of cyclosporin A (CsA), a drug that disrupts the CA-CypA interaction [57]–[59]. To test whether the CsA-dependence observed in A92E mutants is also present in the context of LNEIE, LMNEIE, or INEIE, VSV-G pseudotyped WT and mutant viruses were titrated on HeLa cells in the presence or absence of CsA (Figure S6). As has previously been shown to be the case in HeLa cells [57], the presence of CsA increased infection by the A92E mutant by >10-fold. In contrast, the enhancing effect of CsA on infection was minor in the case of LNEIE (2-fold) and absent in the case of NHG, LMNEIE, and INEIE.

### Lack of rhTRIM5α induced core disruption in rhTRIM5α-resistant viruses

**Figure 6:**
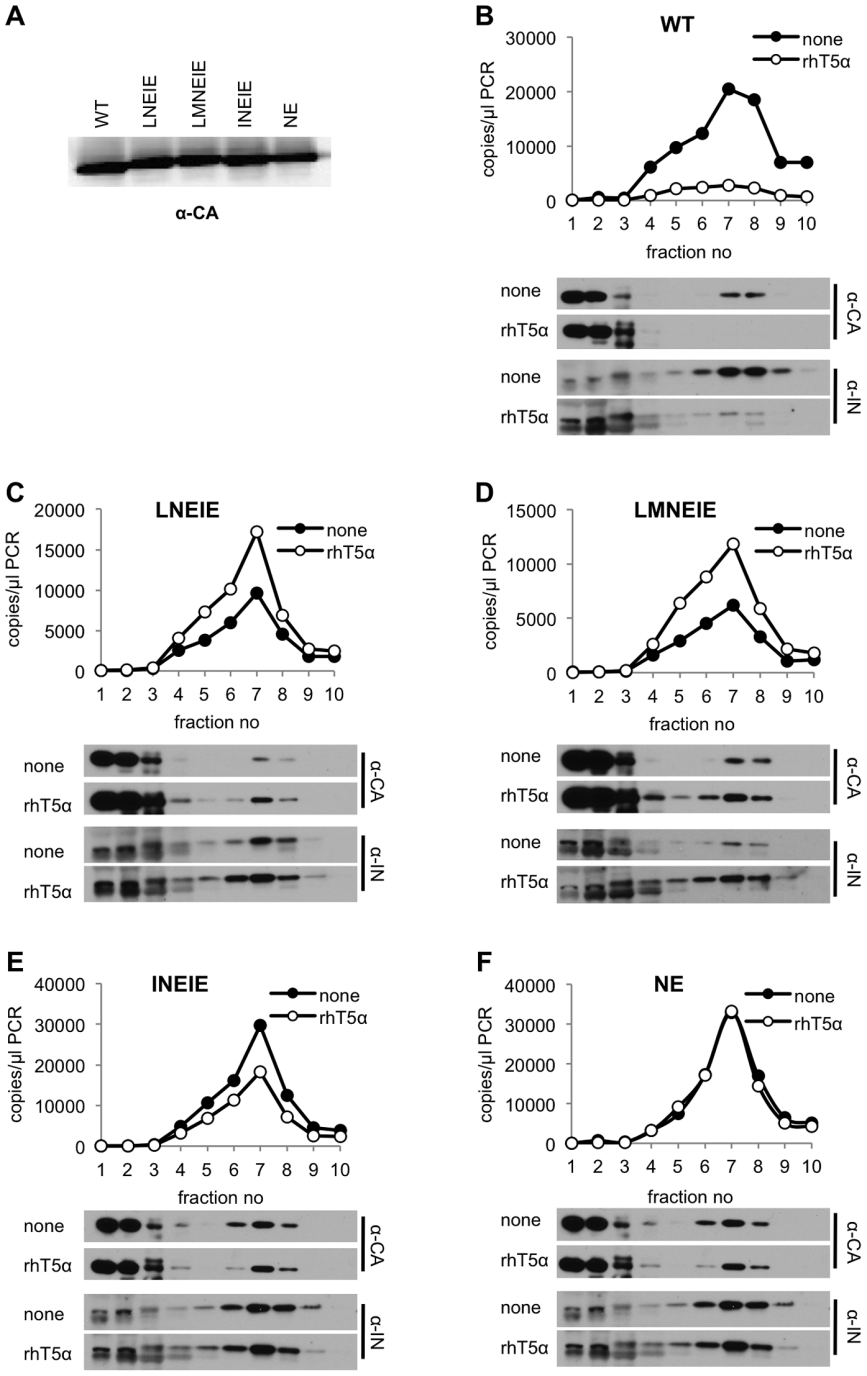
RhTRIM5α-resistant mutants are immune to viral core disruption by rhTRIM5α. (A) Virions harvested from cells transfected with plasmids expressing HIV-1_NL4-3_ GagPol (WT or CA mutants), VSV-G, and an HIV-1 vector carrying a GFP reporter were pelleted through 20% sucrose and CA protein was detected by western blotting. (B–F) PgsA (none) and pgsA-rhTRIM5α (rhT5α) cells were infected with VSV-G pseudotyped HIV-1_NL4-3_ (WT or CA mutants), carrying a GFP reporter. Infected cells were harvested at T = 2 hr. after infection and post-nuclear supernatants were fractionated on 10–50% (w/v) sucrose gradients as described in Materials and Methods. Ten fractions were collected from the gradients. Q-PCR analysis of reverse transcription products and western blot analysis of CA and integrase (IN) in each fraction is represented for HIV-1_NL4-3_ with WT (B), LNEIE (C), LMNEIE (D), INEIE (E) and NE (F) CA sequences.

To further demonstrate resistance of the aforementioned CA mutant viruses to rhTRIM5α, we tested whether viruses carrying the CA mutations were able to avoid the disruptive effects of rhTRIM5α on incoming viral cores [27], [29], [30], and thereby complete reverse transcription. To accomplish this, we employed our recently described assay in which the integrity of a subviral complex containing CA, integrase (IN) and viral nucleic acids is monitored in infected cells shortly after infection [30]. Specifically, VSV-G pseudotyped viruses encoding either WT or mutant CA proteins were generated, and equivalent amounts (Figure 6A) were applied to pgsA-745 or pgsA-rhTRIM5α cells, in a synchronized infection protocol. Two hours later, cytoplasmic extracts were fractionated and the presence of CA and IN protein, as well as viral cDNA was measured in each fraction. As expected, in the case of WT HIV-1, a dense complex containing CA and IN as well as viral DNA was readily detected in infected pgsA cells (Figure 6B). This complex appeared largely absent in identically infected pgsA-rhTRIM5α cells, consistent with the notion that rhTRIM5α disrupts incoming HIV-1 cores. In contrast, the dense complex containing CA and IN, as well as viral DNA, was not disrupted by rhTRIM5α during infection with viruses encoding the LNEIE, LMNEIE, INEIE, or NE CA mutants (Figure 6C–F) and only minor differences in the levels of CA, IN, and viral DNA in the dense complex were observed. These data suggest that the subviral complexes generated by LNEIE, LMNEIE, INEIE, and NE CA mutant viruses are preserved in the presence of rhTRIM5α and thus that the cores of these viruses are largely resistant to the biochemical effects of rhTRIM5α. Although the NE CA mutant retained residual sensitivity to rhTRIM5α in MT2-rhTRIM5α cells (Figure 5C), all four CA mutants (NE, LNEIE, LMNEIE, and INEIE) were able to infect pgsA-rhTRIM5α cells at similar levels (Figure S7). The minor differences among the four mutants in pgsA-rhTRIM5α cells were, apparently, insufficient to be evident in the biochemical assay of rhTRIM5α restriction (Figure 6C–F).

### CA mutations conferring rhTRIM5α resistance prevent recognition by rhTRIM5α

**Figure 7:**
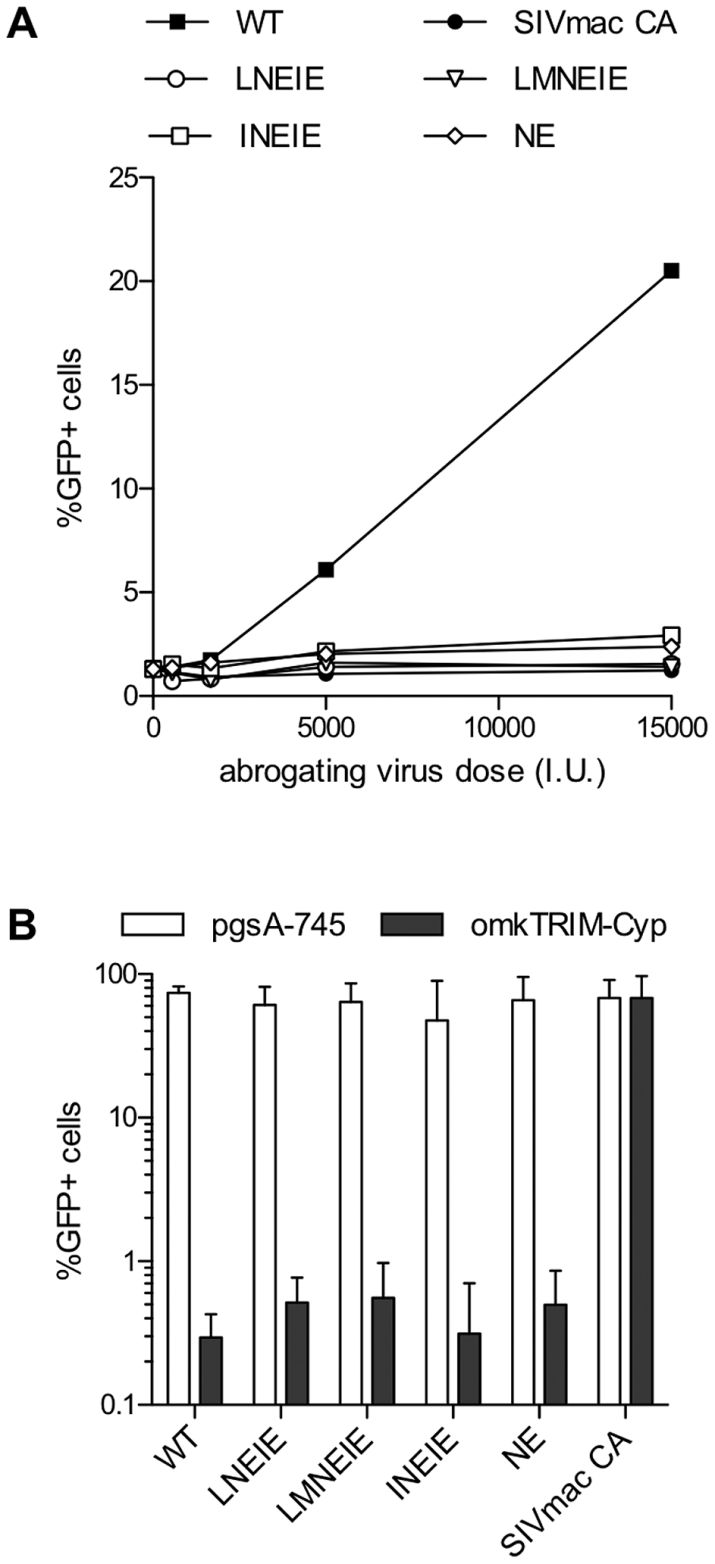
RhTRIM5α-resistant HIV-1 capsids are not recognized by rhTRIM5α. (A) The indicated CA sequences were tested for their ability to generate viral capsids that could saturate rhTRIM5α in a rhesus macaque cell line. FRhK-4 cells were infected with VSV-G pseudotyped virions containing HIV-1_NL4-3_ GagPol proteins encoding either wildtype CA, SIV_mac239_ CA, or the indicated mutant CA. An HIV-1 based vector that encoded Tat was packaged into these virions and ‘abrogating virus dose’ is given in TZM infectious units (I.U.). Cells were tested for rhTRIM5α abrogation by simultaneous infection with a fixed amount of a VSV-G pseudotyped virions carrying a WT HIV-1_NL4-3_ GagPol and a GFP-reporter gene. (B) VSV-G pseudotyped virions carrying the indicated WT or mutant CA-encoding HIV-1_NL4-3_ GagPol and a GFP-reporter gene were used to infect pgsA-745 cells or pgsA-745 cells stably expressing owl monkey TRIM-Cyp. Cells (1×10^4^) were infected with 2 µL of supernatant from transfected 293T cells.

In principle, the above HIV-1 mutants may have acquired resistance to rhTRIM5α by avoiding recognition by rhTRIM5α or, less likely, by acquiring the ability to infect cells despite recognition by rhTRIM5α. To distinguish between these possibilities we performed an ‘abrogation of restriction’ assay. TRIM5α proteins can, in general, be saturated by high amounts of incoming capsids, thereby enabling infection by viruses that would otherwise be restricted [7], [60]. The ability of a given viral capsid to abrogate TRIM5α mediated restriction is thus taken as a surrogate of its ability to be recognized by and bind to that TRIM5α protein. Therefore, we determined whether HIV-1 particles carrying the rhTRIM5α-resistant CA proteins were capable of abrogating rhTRIM5α activity in the rhesus macaque cell line, FRhK-4. VSV-G enveloped HIV-1 particles carrying a minimal HIV-1 genome (lacking GFP) and GagPol proteins that included either WT or the aforementioned rhTRIM5α-resistant CA sequences were first normalized according to their infectious titer on TZM indicator cells. FRhK-4 cells were challenged with increasing amounts of these viral particles along with a fixed amount of a virus containing WT HIV-1 GagPol protein and a genome that encoded a GFP reporter. As expected, infecting FRhK-4 cells in the presence of abrogating HIV-1 virions encoding a WT CA protein dramatically increased the titer of a WT GFP reporter virus (Figure 7A). Notably however, HIV-1 virions encoding rhTRIM5α-resistant CA mutants (NE, LNEIE, INEIE and LMNEIE) failed to abrogate restriction (Figure 7A). the residual sensitivity to rhTRIM5α that was observed for the NE CA mutant in in MT2-rhTRIM5α cells, was barely evident in FRhK4 cells, and all four CA mutants (NE, LNEIE, INEIE and LMNEIE) exhibited only minor variation in infectivity therein (Figure S8). The lack of abrogation activity displayed by the CA mutants strongly suggests that the mechanism by which the CA mutations conferred rhTRIM5α resistance was through loss of specific recognition by rhTRIM5α. Consistent with this conclusion, each of the rhTRIM5α-resistant viruses was inhibited by owl monkey TRIMCyp (Figure 7B), indicating that they were intrinsically sensitive to a TRIM5 protein that is able to bind to these capsids.

### HIV-1 strains encoding rhTRIM5α-resistant CA proteins and SIVmac Vif replicate in rhesus macaque lymphocytes

**Figure 8:**
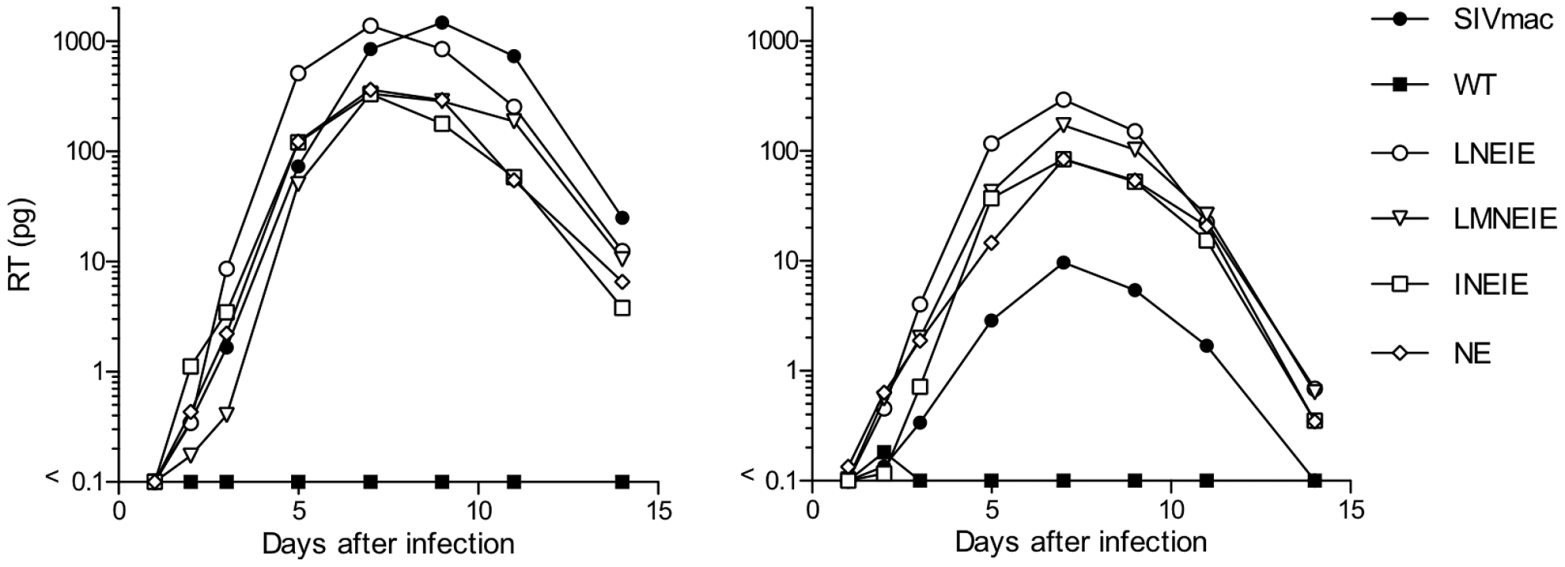
HIV-1 replication in rhesus macaque PBMC. Simian tropic (st) HIV-1 viral stocks encoding SIVmacVif, and either SIV_mac239_ CA, WT HIV-1_NL4-3_ CA, or the indicated mutant HIV-1 CA proteins were normalized according to reverse transcriptase activity and used to infect rhesus macaque PBMC. Viral replication was monitored by measuring the level of reverse transcriptase in the culture supernatant at the indicated times. The two charts show replication assays conducted using PBMC from two different macaque donors.

Along with APOBEC3 proteins, TRIM5α imposes a major block to HIV-1 replication in rhesus macaque cells [39]. To determine whether HIV-1 mutations that conferred resistance to rhTRIM5α expressed in human cell lines enabled replication in primary rhesus macaque cells, the TRIM5α-resistant CA sequences NE, LNEIE, INEIE and LMNEIE were inserted into a chimeric HIV-1 containing SIV_mac239_ Vif, named stHIV-1 [49]. Virus stocks bearing either WT, NE, LNEIE, INEIE, LMNEIE or SIV_mac239_ (stHIV-SCA) [39] CA sequences were normalized for reverse transcriptase content and used to challenge peripheral blood mononuclear cells (PBMC) from two rhesus macaque donors (Figure 8). As expected, stHIV-1 carrying the WT HIV-1 CA sequence failed to replicate, while stHIV-SCA carrying the SIV_mac239_ CA initiated a spreading infection. Notably, all four rhTRIM5α-resistant CA sequences enabled efficient stHIV-1 replication in rhesus macaque PBMC. In one donor, the rhTRIMα-resistant CA mutants replicated similarly to stHIV-SCA while in a second, apparently less permissive donor, all of the CA-mutant HIV-1 strains outperformed stHIV-SCA (Figure 8). The LNEIE mutant appeared to perform marginally better than the other CA mutants, and better than stHIV-SCA in PBMC from both donors. Genotyping revealed that both donors were heterozygous for rhTRIM5α alleles. The first donor carried alleles 4 and 5, while the second carried alleles 3 and 4 as (following the nomenclature described by Newman *et al* and Wilson *et al* [54], [55]). Alleles 4 and 5 belong to a class that encodes a glutamine at residue 339 and allele 3 belongs to a class that encodes TFP at the same position. These two classes of rhTRIMα variants have been shown to differ in their restriction specificity and potency [54], [55]. Importantly, the ability of the HIV-1 mutants described here to replicate in PBMC from both animals suggests that they have acquired resistance to restriction by both classes of rhTRIM5α alleles.

## Discussion

In this study, we identified a number of mutations in HIV-1 CA that individually could reduce the sensitivity of the incoming capsid to restriction by rhTRIM5α. When present in the right combination, collections of these mutations could confer near complete resistance to rhTRIM5α, sometimes without a fitness cost. Indeed, mutations in capsid were necessary and sufficient for HIV-1 to evade restriction by rhTRIM5α, consistent with the notion that the antiviral activity of TRIM5 depends on specific capsid recognition [5], [8], [39], [40], [47]. The rhTRIM5α resistant CA sequences abolished the ability of rhTRIM5α to disrupt incoming HIV-1 cores, enabling reverse transcription and the formation of a complex containing CA, IN and viral DNA, which would normally be blocked by rhTRIM5α [30]. Ultimately, these mutations enabled uninhibited infection of human cell lines stably expressing rhTRIM5α, which ordinarily exhibit >50-fold reduced susceptibility to WT HIV-1 infection.

With one exception, the amino acid substitutions that were found in the rhTRIM5α-resistant CA sequences encoded amino acids that are exposed on the presumptive exterior surface of the capsid lattice. However, L52I, (which occurred in only one of two cell lines in which the assorted CA mutant pool was evolved) is buried in the interior of the CA protein structure [43]. It is possible that L52I contributes to resistance by shifting the conformation of the capsid surface. Alternatively, it may marginally stabilize or destabilize the viral core, thereby affecting rhTRIM5α action. It is also possible that L52I, or indeed other mutations described herein, arose as a compensatory mutation to maintain high viral fitness while not directly contributing to rhTRIM5α resistance. The contribution of the L52I mutation in the interior of the CA structure notwithstanding, the primary mechanism by which the HIV-1 CA mutants acquired resistance to rhTRIM5α appeared to be through loss of rhTRIM5α recognition, rather than resistance to the effects of TRIM5α after recognition of the incoming capsid. This conclusion is based on the findings that (i) rhTRIM5α-resistant mutant capsids were unable to saturate rhTRIM5α and thereby facilitate WT HIV-1 infectivity in rhesus macaque cells, suggesting that they were not recognized by rhTRIM5α and (ii) the rhTRIM5α-resistant capsids retained full sensitivity to another TRIM5 protein (omkTRIMCyp) with a different CA binding specificity. Neither of these results would be expected if resistance were acquired via a mechanism in which the viral capsid retained rhTRIM5α binding, but acquired the ability to resist its antiviral effects.

Our initial failure to derive HIV-1 variants with complete resistance to rhTRIM5α through replication in cell lines expressing this inhibitor, or through random mutagenesis, underscores the difficulty in deriving HIV-1 strains with this property. Indeed, our eventual success required the combined application of the two different approaches, and then further evolution during selection from an assortment of mutations identified by each strategy. A comparison of the results obtained from the initial adaptation (Figure 2) and random mutagenesis (Figure 3) approaches suggests that each had distinct advantages and disadvantages. Despite the fact that four parallel cultures were initiated, the adaptation approach clearly did not produce a wide variety of solutions, and the same four mutations (V86E, I91N, I91T, and G116E) arose in four independent cultures. No combination of these four mutations gave fully fit rhTRIM5α-resistant CA sequences. It is possible that this result is defined by a potentially limited complexity of the viral population to which selection pressure was applied, and that rather than selection of several independent solutions, a small number of initially dominant genotypes persisted. However, another explanation might be that mutations that reduce HIV-1 sensitivity to rhTRIM5α without a fitness cost are few in number. Consistent with this latter interpretation is the fact that 2 of the 3 positions that we found to be mutated in rhTRIM5α-selected viruses have also been identified in similar, but completely independent, selection experiments in other laboratories, in different cell lines [44], [45]. Those mutations that confer rhTRIM5α resistance but are marginally deleterious to virus replication might not be selected during the adaptation approach, even though they could contribute rhTRIM5α evasion if their accompanying fitness costs were alleviated by compensating mutations. Screening a random library of virus clones might be a better way to identify such mutants. Indeed, the random mutagenesis screen identified a larger number of mutants that conferred reduced rhTRIM5α sensitivity, even though many of these did have associated fitness defects. Notably, V86E and I91T emerged from both adaptation and the random mutant library screening approaches. Interestingly, however, the random mutagenesis screen identified several mutations that did not arise during adaptation yet exhibited reduced sensitivity to rhTRIM5α without an obvious fitness cost (e.g. V83M and M10L). Nevertheless, a drawback of random mutant screens is that only a limited number of mutants can be individually tested. Indeed, two amino acid substitutions emerged during *in vitro* evolution that were not represented in the random mutant library (G116E and I91N). Additionally, because the experiments were done in human cells, a potential limitation of both the random mutant library screening and the *in vitro* evolution strategies was the possibility that some rhTRIM5α-resistant mutants could be missed if they simultaneously caused gain of sensitivity to endogenous human TRIM5α.

Overall, however, the application of both approaches and assortment of the resulting mutants in an assisted evolution approach led to derivation of fit, rhTRIM5α-resistant CA sequences. Even then, further *de novo* mutations of the assorted variant pool was required to generate the optimally resistant CA sequences. One possible reason for the eventual success of our approach is that the second round of selection was performed using a population of CA sequences that was highly enriched for mutations conferring partial TRIM5α resistance. This population contained individual mutant assortants that are highly unlikely to have occurred by chance through the standard approaches to viral evolution that were attempted initially.

Clearly, each individual mutation identified by either adaptation or random mutant screening approaches enabled only a partial evasion of rhTRIM5α (Figures 2 and 3). These mutations were distributed over several determinants on the surface of the HIV-1 capsid, as has been found in previous studies of retrovirus sensitivity to TRIM5α [40], [41], [44]–[47]. Indeed, although the cyclophilin-binding loop was featured prominently as a site at which mutations conferring decreased HIV-1 sensitivity to rhTRIM5α occurred (positions L83, V86, I91, A92, M96), other determinants included the N-terminal β-hairpin (M10) and helix 6 (G116). One reasonable interpretation of these data is that several different sites on the capsid exterior contribute to the binding interaction with TRIM5α, and that mutations at any one of these sites, reduces, but does not eliminate interaction. Interestingly, a comparison of the distribution of mutations in MLV CA and HIV-1 CA that arose during the selection of rhTRIM5α-resistant viral variants [47] reveals striking similarity (Figure S9A and S9B). A key difference, however, is that single amino acid substitutions in MLV CA conferred near complete resistance to rhTRIM5α [47], while multiple substitutions were required in HIV-1 to achieve the same effect. Perhaps the viral challenges to which rhTRIM5α has been subjected to during its evolutionary history have shaped it in such a way that it is a more robust inhibitor of lentiviruses than gammaretroviruses.

Because mutations in the cyclophilin-binding loop of HIV-1 CA have previously been shown to reduce the contribution of CypA to rhTRIM5α activity [61], [62], it is possible that perturbation of CypA binding may have contributed to the acquisition rhTRIM5α resistance described herein. Although retention of sensitivity to restriction by TRIM-Cyp suggests that CypA binding has been retained in the mutants described herein (Figure 7B), it remains possible that the role of CypA in rhTRIM5α activity was perturbed. Indeed previous work has suggested that a V86M CA mutation, while not preventing CypA binding, eliminates the contribution of CypA to the restriction of HIV-1 by huTRIM5α mutants [63]. It therefore remains unclear whether cyclophilin-binding loop mutations in rhTRIM5α-resistant capsids have altered the involvement of CypA in restriction or simply decrease the binding of rhTRIM5α to HIV-1, independent of CypA.

Although HIV-1 CA mutations could be combined to give near complete escape from restriction, some combinations, particularly those derived from the first round of selection, were apparently deleterious to viral fitness. The need to alter multiple determinants, coupled with the inherent genetic fragility of HIV-1 CA [48] likely underlies the difficulty in generating combinations of CA mutations that confer rhTRIM5α resistance while maintaining high fitness. It is possible that the LNEIE and INEIE mutants, which were both fit and resistant to restriction by rhTRIM5α, include compensatory changes that alleviate fitness costs that accompanied escape from rhTRIM5α restriction. Our group has recently demonstrated that about 70% of randomly introduced single amino acid substitutions, and nearly all two-amino acid substitutions in CA are lethal to HIV-1 [48]. This lack of robustness in CA is also evident in MLV, as the N-terminal domain of MLV CA, unlike other regions of Gag, is intolerant of small insertions [64]. In the case of HIV-1, most lethal CA mutations were shown to disrupt normal particle formation [48]. However, interactions with multiple host factors, or structural requirements that dictate proper disassembly may also decrease the genetic robustness of CA. Thus, mutants containing combinations of changes required to both escape rhTRIM5α and retain fitness should be very rare, and this may be one reason why evolution has selected CA as a target for host antiviral proteins. Notably, the incomplete escape that was evident in viral populations derived from initial selection experiments included a double amino acid mutant (NE), which was both fit and incompletely resistant to restriction in MT2-rhTRIM5α cells, as well as mixtures of viruses containing WT and mutant codons at V86 and G116. Although a V86E/G116E double mutant clearly had the opportunity to dominate in these cultures, it exhibited a clear loss of fitness, probably explaining why it did not. While it is formally possible that the fitness defect in V86E/G116E and other unfit rhTRIM5α-resistant mutants is species-specific and would not exist in monkey cells, we consider this unlikely, given that low infectiousness was also observed in hamster cells (Figure S2).

Importantly, mutations conferring resistance in rhTRIM5α-expressing cell lines allowed HIV-1 encoding SIV_mac239_ Vif to replicate in PBMC from rhesus macaques. Indeed, an HIV-1 encoding the LNEIE mutant CA protein replicated as well as or better than a virus encoding an SIV_mac239_ CA protein, suggesting that TRIM5α activity was entirely bypassed. Therefore, the HIV-1 CA mutations identified here enable HIV-1 to overcome the species-specific tropism barrier imposed by rhTRIM5α. Thus, although the barrier to the evolution of fit, rhTRIM5α-resistant HIV-1 CA proteins is apparently high, it is not insurmountable. Moreover, although the two amino acid NE mutant was inferior to the 5 and 6 amino acid CA mutants, in that it retained some degree of sensitivity to rhTRIM5α in human cell lines, the NE mutant replicated well in rhesus PBMC, and had little if any ability to saturate rhTRM5α in the FRhK cell line. Thus, one caveat that should be attached to the above discussion is that the level of rhTRIM5α expression in the engineered human cell lines in which our selection experiments were performed likely exceeds the endogenous levels present in rhesus macaque cells. The number of mutations required to operationally escape TRIM5α inhibition in a natural setting may therefore be fewer than is suggested by our studies. In any case, the CA mutants identified herein should facilitate the development of rhesus macaque-based non-human primate models of HIV-1 infection.

## Materials and Methods

### Cell lines and viruses

Primate adherent cells (293T and FRhK-4) and suspension cells (MT2, MT4, CEM, and rhesus macaque PBMC) were cultured in DMEM and RPMI, respectively. CHO K1-derived pgsA-745 and the previously described pgsA-rhTRIM5α and pgsA-omkTRIMCyp cell lines [30] were maintained in Ham's F12 media with 1 mM L-glutamine. All growth media was supplemented with 10% fetal bovine serum and gentamicin.

Human T-cell lines (MT2, MT4 and CEM) expressing rhTRIM5α were prepared by transduction with a murine leukemia virus (MLV) vector containing rhTRIM5α cDNA, inserted 5′ to an internal ribosomal entry site and a blasticidin resistance gene. The rhTRIM5α cDNA was previously cloned from FRhK-4 cells [3] and the sequence matches allele 3, as described by Lim *et al* [65]. This rhTRIM5α allele belongs to a class of rhTRIM5α alleles that encode TFP at amino acid residues 239–241 and restrict HIV-1 more potently than other alleles [54], [55]. To generate the cell lines, 293T cells were cotransfected using polyethyleneimine (PolySciences) as previously described [7] with 100 ng of a vesicular stomatitis virus G protein (VSV-G) expression plasmid, 500 ng of a MLV GagPol expression plasmid, and 500 ng of the rhTRIM5α-expressing MLV vector. The resulting virus was used to transduce MT2, MT4, or CEM cells. Cells were selected with 10 µg/mL blasticidin and single-cell clones expressing rhTRIM5α were derived by limiting dilution.

The replication competent HIV-1/GFP used in viral evolution experiments and for measurements of rhTRIM5α sensitivity was produced by transfection of an HIV-1_NL4-3_/HIV-1_HXB2_ recombinant proviral plasmid that encoded GFP in place of Nef (pNHG, GenBank accession number: JQ585717) [66]. Infectious titer was determined by inoculating MT2 cells (2×10^4^ per well) in 96-well plates with 50 µL of serially diluted supernatant. After allowing infection to proceed overnight, further replication was blocked by the addition of 100 µg/mL dextran sulfate. Two days after infection, cells were fixed in 2% paraformaldehyde and the number of infected cells enumerated by FACS.

Viruses used to saturate rhTRIM5α in FRhK-4 cells were prepared by cotransfection of 293T cells with pCRV1-HIV-1_NL4-3_ GagPol (WT or rhTRIM5α-resistant mutant), the HIV-1 based vector pV1, which lacks a reporter gene, and a VSV-G expression plasmid at a 5∶5∶1 ratio, respectively [16]. Titers were determined using TZM-bl indicator cells, infected colonies were stained and counted using X-Gal two days post-infection. The reporter virus used to test for rhTRIM5α saturation in FRhK-4 cells, the viruses used for the biochemical analysis of core components, and the viruses used to infect pgsA-745 cell lines were produced in the same manner, except an HIV-1 based vector encoding GFP (pCSGW) [67] was used instead of pV1. VSV-G enveloped N-MLV was also produced in the same manner, except N-MLV GagPol expression plasmid and an MLV based vector encoding GFP (pCNCG) were used [68].

### Virus evolution in rhTRIM5α expressing cells

Prior to inoculation of MT2 and MT4 cells expressing rhTRIM5α, a cloned pNHG-derived virus that lacked Vpr was allowed to replicate in MT2 cells to generate sequence diversity. Specifically, 5×10^5^ MT2 cells were infected with NHG at an MOI of 0.001. To maintain a viable cell population (the culture would have otherwise died due to cytopathic effects) and expand the viral population, uninfected MT2 cells were added as follows: 5×10^5^ cells at 4 dpi, 10^6^ cells at 6 dpi and 8 dpi, 3×10^6^ cells at 10 dpi, and 4×10^6^ cells at 13 dpi. After 15 days of replication, cell-free supernatant was harvested and titrated on MT2 cells. Thereafter, 1.25×10^6^ IU were used to infect two cultures of 5×10^6^ MT2-rhTRIM5α cells, two cultures of MT4-rhTRIM5α cells, and one culture each of MT2 and MT4 cells transduced with empty vector. Cells were diluted to 35 mL after an overnight infection in 5 mL. To measure the initial infection, dextran sulfate was added to a 100 µL aliquot of cells one day after infection, which were then fixed the following day. To monitor the spreading infection, 100 µL aliquots of cells were fixed periodically in 2% paraformaldehyde and the fraction of infected (GFP positive) cells determined by FACS analysis. After cytopathic effects became abundant in the culture, and/or the percentage of infected cells had peaked, 2 mL cell-free supernatant was used to infect 5×10^6^ fresh cells as before. However, beginning with the fifth passage in MT2- rhTRIM5α cell lines and the fourth passage in MT4- rhTRIM5α cell lines 5 mL of supernatant was used to initiate new passages.

To recover CA sequences from viruses replicating in rhTRIM5α expressing cells, DNA was isolated from infected cells using the Qiagen DNeasy Blood and Tissue Mini Kit and sequences encoding CA were amplified using PCR and a sense primer, 5′-AAGGAAGCCTTAGATAAGATAGAGG-3′ along with an antisense primer 5′- TTGCCTTTCTGTATCATTATGGTAG -3′. Products were directly sequenced, without cloning, using the sense primer. To generate proviral clones containing adapted CA sequences, PCR amplification was done using primers 5′-GCACAGCAAGCGGCCGCTGACACAGG-3′ and 5′-GCCAAAACGCGTGCTTTATGGCCGGG-3′ to add NotI and MluI restriction sites for insertion into a pNHG derivative that was engineered (by silent mutagenesis) to contain NotI and MluI restriction sites flanking CA (GenBank accession number JQ686832). Additional combinations of mutations were introduced into CA by PCR-based mutagenesis methods as previously described [69]. For follow-up studies of CA mutants, virus stocks were generated by transfection of 293T cells with 1 µg proviral plasmid/well in a 24-well plate. Reverse transcriptase in culture supernatants was quantified by a previously described assay based on real-time PCR [70], and infectious titers were determined as described above. Some CA mutant viruses were also used in spreading replication experiments, in which 2×10^5^ MT2-rhTRIM5α cells or empty vector transduced MT2 cells in 12-well plates were infected at an MOI of 0.002. Culture volumes were kept at 1 mL throughout the experiment. One day after infection, a 100 µL aliquot of cells was treated with 100 µg/mL dextran sulfate and fixed the following day to measure the initial infection. Beginning two days after infection, 100 µL aliquots of cells were fixed daily for quantitation of GFP positive cells by FACS analysis.

### Screen of random HIV-1 CA mutants for rhTRIM5α resistance

The construction and analysis of a library of pNHG clones encoding PCR-mutagenized CA sequences has been previously described [48]. Briefly, the mutagenized CA library was generated using the Genemorph II random mutagenesis kit (Agilent) using the following oligos 5′-GTA AGA AAA AGG CAC AGC AAG CGG CCG CTG -3′ and 5′- CTT GGC TCA TTG CTT CAG CCA AAA CGC GTG-3′. The PCR template was a pNHG derivative (JQ686832) that contained silently mutated sequences to generate *Not*I and *Mlu*I sites flanking CA-encoding sequences (pNHGCapNM). The PCR product was cloned using a TOPO TA Cloning Kit and plasmid DNA was extracted from approximately ∼1×10^4^ pooled, insert-positive colonies. After sequencing the amplicon from 20 clones to obtain a preliminary estimate of the mutagenesis frequency, this pooled plasmid DNA was digested using *Not*I and *Mlu*I and the CA-library insert subcloned into pNHGCapNM. Proviral plasmid DNA was extracted from individual cultures derived from 1056 colonies and subjected to sequencing and further analysis. Ninety-one of the resulting proviral plasmids that yielded infectious virions were screened for mutations that decrease sensitivity to restriction by rhTRIM5α. Specifically, viral stocks containing WT NHG or each of the 91 CA mutants were prepared by transfecting 2.5×10^4^ 293T cells/well with 100 ng of pNHG and polyethylenimine in a 96-well plate. Thereafter, MT2-rhTRIM5α cells or MT2 cells transduced with empty vector were infected with 2 µL filtered virus in a 96-well plate at 2×10^4^ cells/well. Virus replication was limited to a single cycle by adding 100 µg/mL dextran sulfate after an overnight infection. Two days after infection, cells were fixed in 2% paraformaldehyde and subjected to FACS analysis. Stocks of 22 mutants that infected at least 0.1% MT2-rhTRIM5α cells and were restricted by less than 50-fold (see results) were prepared again by transfecting 2×10^5^ 293T cells with 1 µg pNHG in a 24-well plate. Again, single-cycle infections were done in MT2-rhTRIM5α cells or MT2 cells transduced with empty vector, using 50 µL of serially diluted supernatant.

### Construction and evolution of a library of synthetic CA sequences containing mutant assortments

To construct a library of CA sequences containing random assortments of CA mutations (M10L/V, L83M, V86E, H87Q, I91N/T, R100S, A105T, and G116E), ten overlapping oligonucleotides, four containing degenerate nucleotides, were used in a PCR-based gene synthesis to produce a 400 base pair DNA encoding the 5′ portion of CA that included all of the mutated positions. The heterogeneous gene synthesis product was designed to encode WT or mutant amino acids at a 1∶1 ratio, or a 1∶1∶1 ratio in cases where two mutations were included at a single position. Specifically, a 50 µL PCR reaction contained 3 pmol of each oligonucleotide and gene synthesis was carried out for 20 cycles using Phinzymes Phusion (1 min. 98°C, 1 min. 50°C, and 2 min. 72°C). Thereafter, 2 µL of crude gene synthesis product was used as template in a 50 µL PCR reaction with 200 nM external primers (1 min. 98°C, 1 min. 55°C, and 2 min. 72°C) and the resulting product was gel-purified. The 5′ end of the gene synthesis product was designed to contain a Not I restriction site while the 3′ end was designed to overlap with a second amplicon that was used to extend the synthetic CA sequence to include the 3′ portion of CA and an MluI restriction site. The two overlapping DNAs were used together as templates for a final PCR, which produced a DNA that was gel purified, and inserted into pNHG. Proviral plasmid DNA was purified from 2.5×10^3^ pooled bacterial colonies and 5 µg of this DNA was used to transfect 293T cells. The resulting virus was used to infect 5×10^6^ MT2-rhTRIM5α cells at an MOI of 0.005. Then, 1 mL of cell-free virus was used in a second passage onto MT2-rhTRIM5α cells and 100 µL was used in all subsequent passages. Following 11 passages in MT2-rhTRIM5α cells, 2 mL of cell-free supernatant was used for serial passaging in CEM-rhTRIM5α cells.

### Biochemical analysis of retroviral cores

Biochemical analysis of retroviral cores in infected cells was done as previously described [30]. Briefly, pgsA745 cells or its derivative stably expressing rhTRIM5α were plated in 10-cm dishes one-day before infection. Cell culture supernatants containing VSV-G pseudotyped HIV-1_NL4-3_ GagPol/CSGW viruses were filtered and treated with RNase free DNaseI (Roche) at a concentration of 1 unit/ml for 1 hour at 37 °C in the presence of 6 mM MgCl_2_. Cells were washed with ice-cold phosphate-buffered saline (PBS) and 7 ml of chilled virus (with 20 mM HEPES) was added to the cells at an MOI of ∼0.01. After allowing virus to bind to cells for 30 minutes at 4°C, the inoculum was removed and cells were washed three times with PBS. Cells were then incubated at 37°C for 2 hours in complete cell culture media. Cells were collected in PBS-EDTA, pelleted, and resuspended in 1 ml of hypotonic buffer (10 mM Tris-Cl pH 8.0, 10 mM KCl, 1 mM EDTA) supplemented with complete protease inhibitors (Roche) and SuperaseIN (Life Technologies). After incubation on ice for 15 minutes, the cell suspension was dounce homogenized for 50 strokes, using pestle B. Nuclear material was pelleted by centrifugation at 1000×*g* for 5 minutes and post-nuclear supernatant was layered on top of a 10–50% (w/v) linear sucrose gradient prepared in 1X STE buffer (100 mM NaCl, 10 mM Tris-Cl pH 8.0, 1 mM EDTA). Samples were ultracentrifuged using a SW50.1 rotor at 30000 rpm for 1 hour. Ten 500 µl fractions from the top of the gradient were collected, and proteins and DNA in each fraction were analyzed as previously described [30].

### Virus replication in rhesus macaque PBMC

Rhesus macaque PBMC were stimulated with 3 µg/mL staphylococcus enterotoxin B (SEB) for three days and 5% IL-2 (Hemogen) throughout the experiment. Following SEB stimulation, PBMC were infected with stHIV-1, which is a chimeric HIV-1_NL4-3_ –derived virus in which the Env protein is derived from SHIV/KB9 and the Vif protein is replaced by that of SIV_mac239_. Additionally, stHIV-1/S-CA encodes an SIV_mac239_ CA sequence [39], [49]. Mutant HIV-1_NL4-3_ CA sequences were transferred from NHG to stHIV-1, using BssHII and AgeI restriction sites. Virus stocks were normalized for reverse transcriptase activity and 1×10^5^ PBMC were infected in a 96-well V-bottom plate with inocula containing 20 pg reverse transcriptase. Following overnight infection, cells were washed three times and supernatant from the third wash was frozen for RT quantitation (this value is plotted as 1 day after infection). After washing, cells were transferred to a 96-well U-bottom pate. At days 2, 3, 5, 7, 9, 11, and 14 after infection 80% of the cell supernatant was frozen for RT quantitation and replaced with fresh medium. For rhTRIMα genotyping, DNA was isolated from PBMC from both donors using the QIAGEN DNeasy Blood & Tissue Mini Kit. RhTRIMα exon 8 was amplified by PCR using sense primer 5′- TTGATGTGACACTGGCTCCAAACAAC-3′ and antisense primer 5′-TGGGTAAAGCGGCCGCCAGAGCTTG-3′. The PCR products were cloned using the Invitrogen Zero Blunt TOPO PCR Cloning Kit.

## Supporting Information

Figure S1 **Replication of HIV-1 carrying combinations of CA mutations that reduce sensitivity to rhTRIM5α.** NHG (WT) or NHG derivatives carrying the indicated mutations were used to infect MT2-vector (open symbols) or MT2-rhTRIM5α#8 (filled symbols) cells. All infections were done at an equivalent MOI. To monitor the spreading infections, aliquots of cells were fixed daily for FACS analysis. The percentage of cells expressing GFP is plotted against the number of days after infection.
(TIF)

Figure S2 **Attenuated CA mutants did not acquire sensitivity to human TRIM5α.** PgsA-745 cells (open symbols) or pgsA-745 cells stably expressing human TRIM5α (filled symbols) were infected with VSV-G pseudotyped virions that carried a GFP-reporter gene and WT or mutant CA sequences in the context of HIV-1_NL4-3_ GagPol, as indicated. A VSV-G enveloped virus carrying N-tropic MLV GagPol and a GFP-reporter gene was also included as a positive control for human TRIM5α activity. The infectious titers on each cell line are plotted (circles, referencing the left Y-axis) and the ratio of the titers is shown as fold restriction (triangles, referencing the right Y axis).
(TIF)

Figure S3 **Vertical mutagenesis at positions where mutations arose during passage in rhTRIM5α-expressing cells.** (A–C) MT2-vector (open symbols) or MT2-rhTRIM5α#8 (filled symbols) were infected with WT NHG or NHG encoding the amino acids indicated on the X axis at positions V86 (A), I91 (B) or G116 (C). Each mutant encoded a single amino acid substitution, and the panel covered each of the 19 possible amino acid substitutions at each position. Infectious titers (circles, referencing the left Y-axis) and the ratio of the titers (fold restriction, red triangles referencing the right Y-axis) are plotted. WT NHG is the leftmost data point on each chart.
(TIF)

Figure S4 **Some mutations at CA positions V86, I91 and G116 have modest effects on sensitivity to human TRIM5α.** (A–C) PgsA-745 cells (open circles) or pgsA-745 cells stably expressing human TRIM5α (filled circles) were infected with VSV-G pseudotyped virions that carried a GFP-reporter gene and HIV-1_NL4-3_ GagPol encoding WT or mutant CA sequences. Amino acid substitutions at positions V86 (A), I91 (B), or G116 (C) are indicated on the X axis as in Figure S2. Infectious titers on each cell line (circles, referencing the left Y-axis) and the fold difference in the two titers (triangles, referencing the right Y-axis) are plotted. N-MLV was included as a positive control for restriction (C, rightmost data point).
(TIF)

Figure S5 **Resistance to rhTRIM5α in spreading replication assays.** NHG (WT) or NHG derivatives carrying the indicated mutations were used to infect MT2-vector (open symbols) or MT2-rhTRIM5α#8 (filled symbols) cells at the same MOI. To monitor the viral spread, aliquots of cells were fixed daily for FACS analysis. The percentage of infected (GFP positive) cells is plotted against the number of days after infection.
(TIF)

Figure S6 **The CsA-dependent phenotype exhibited by the A92E CA mutant is not exhibited by the LNEIE, LMNEIE, or INEIE CA mutants.** VSV-G pseudotyped NHG encoding WT or mutant CA sequences was used to infect HeLa cells in the absence (open symbols) or presence (filled symbols) of 5 µM CsA, as previously described [57]. The percentage of infected (GFP positive) cells is plotted versus the volume of each inoculum.
(TIF)

Figure S7 **Infectivity of NE, LNEIE, LMNEIE, and INEIE CA mutants in pgsA-rhTRIM5α cells.** PgsA-745 (left) or pgsA-rhTRIM5α cells (right) were infected with VSV-G pseudotyped virions that carried a GFP-reporter gene and HIV-1_NL4-3_ GagPol encoding WT or the indicated mutant CA sequences. The percentage of GFP positive cells is plotted for each virus dose.
(TIF)

Figure S8 **Infectivity of NE LNEIE, LMNEIE, and INEIE CA mutants in FRhK-4 cells.** VSV-G pseudotyped virions that carried a GFP-reporter gene and HIV-1_NL4-3_ GagPol encoding WT or the indicated mutant CA sequences were used to infect FRhK-4 cells. The percentage of cells infected (GFP positive) is plotted as a function of virus dose.
(TIF)

Figure S9 **A comparison of the distribution of mutations in MLV CA and HIV-1 CA that reduce sensitivity to rhTRIM5α.** (A) Positions of amino acid residues mutated in the best performing HIV-1 CA mutant (LNEIE: M10, I91, A92, M96, G116) are indicated in green on the HIV-1_NL4-3_ capsid hexameric structure. (B) Positions of individual amino acid residues that confer complete resistance to rhTRIM5α (L10, H114, E92) [47] are indicated on the MLV CA NTD hexameric structure.
(TIF)
