# Supplementary figures and images for: Assisted Evolution Enables HIV-1 to Overcome a High TRIM5α-Imposed Genetic Barrier to Rhesus Macaque Tropism
Source: PLoS Pathog. 2013 Sep 26;9(9):e1003667. doi: 10.1371/journal.ppat.1003667 (PMC3784476; doi:10.1371/journal.ppat.1003667)

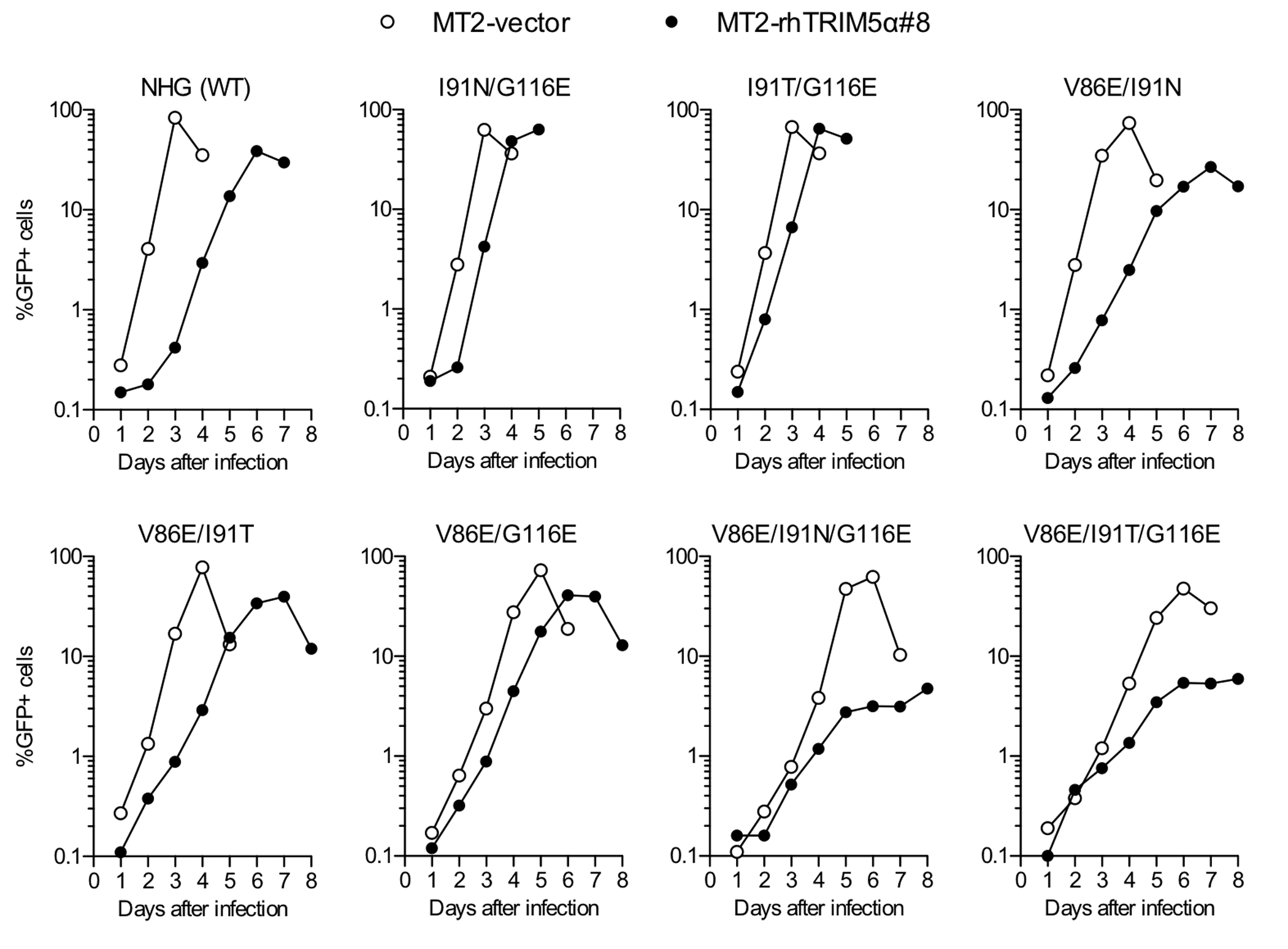

Supplement: Figure S1 — Replication of HIV-1 carrying combinations of CA mutations that reduce sensitivity to rhTRIM5α. NHG (WT) or NHG derivatives carrying the indicated mutations were used to infect MT2-vector (open symbols) or MT2-rhTRIM5α#8 (filled symbols) cells. All infections were done at an equivalent MOI. To monitor the spreading infections, aliquots of cells were fixed daily for FACS analysis. The percentage of cells expressing GFP is plotted against the number of days after infection. (TIF) [file ppat.1003667.s001.tif]

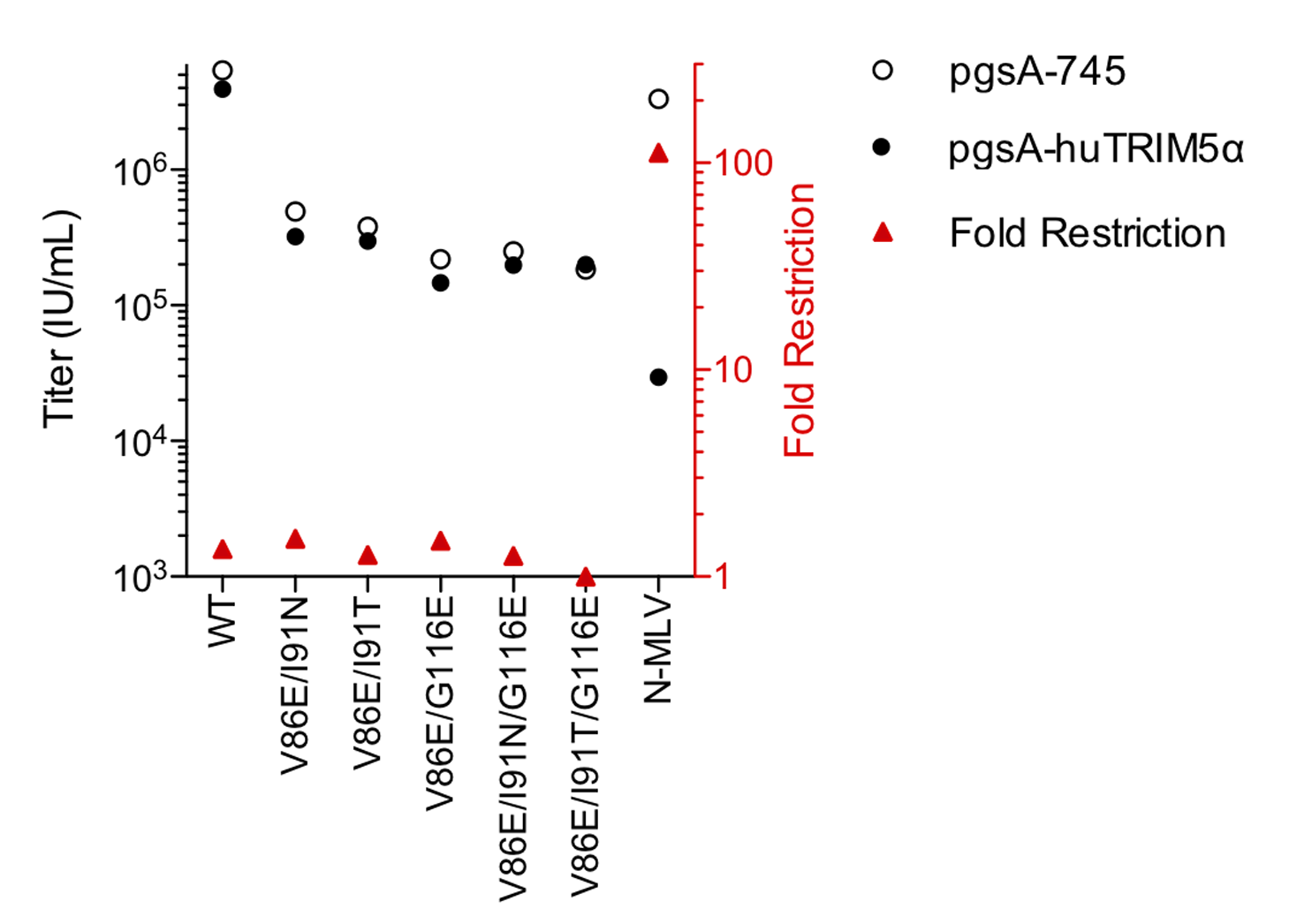

Supplement: Figure S2 — Attenuated CA mutants did not acquire sensitivity to human TRIM5α. PgsA-745 cells (open symbols) or pgsA-745 cells stably expressing human TRIM5α (filled symbols) were infected with VSV-G pseudotyped virions that carried a GFP-reporter gene and WT or mutant CA sequences in the context of HIV-1NL4-3 GagPol, as indicated. A VSV-G enveloped virus carrying N-tropic MLV GagPol and a GFP-reporter gene was also included as a positive control for human TRIM5α activity. The infectious titers on each cell line are plotted (circles, referencing the left Y-axis) and the ratio of the titers is shown as fold restriction (triangles, referencing the right Y axis). (TIF) [file ppat.1003667.s002.tif]

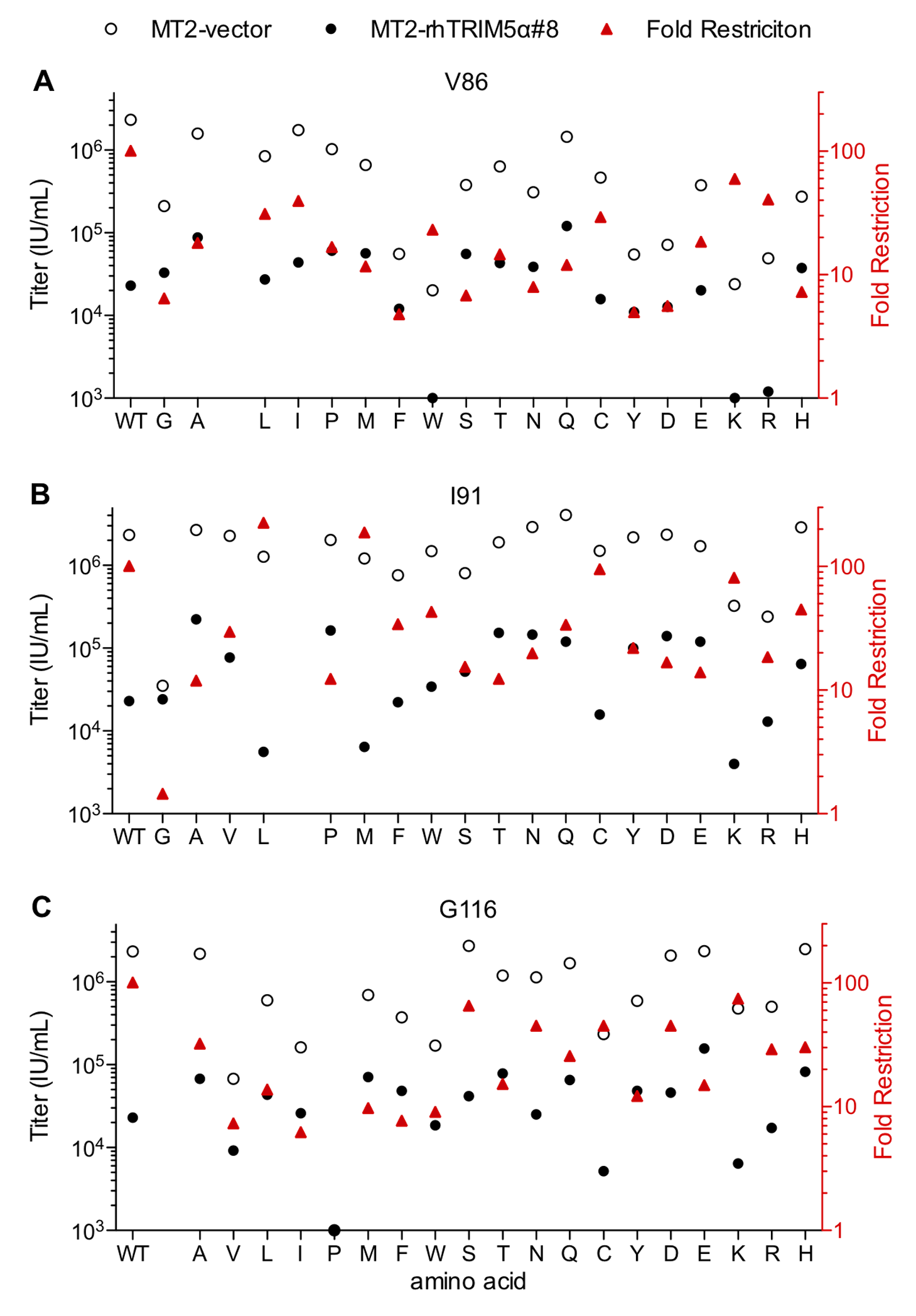

Supplement: Figure S3 — Vertical mutagenesis at positions where mutations arose during passage in rhTRIM5α-expressing cells. (A–C) MT2-vector (open symbols) or MT2-rhTRIM5α#8 (filled symbols) were infected with WT NHG or NHG encoding the amino acids indicated on the X axis at positions V86 (A), I91 (B) or G116 (C). Each mutant encoded a single amino acid substitution, and the panel covered each of the 19 possible amino acid substitutions at each position. Infectious titers (circles, referencing the left Y-axis) and the ratio of the titers (fold restriction, red triangles referencing the right Y-axis) are plotted. WT NHG is the leftmost data point on each chart. (TIF) [file ppat.1003667.s003.tif]

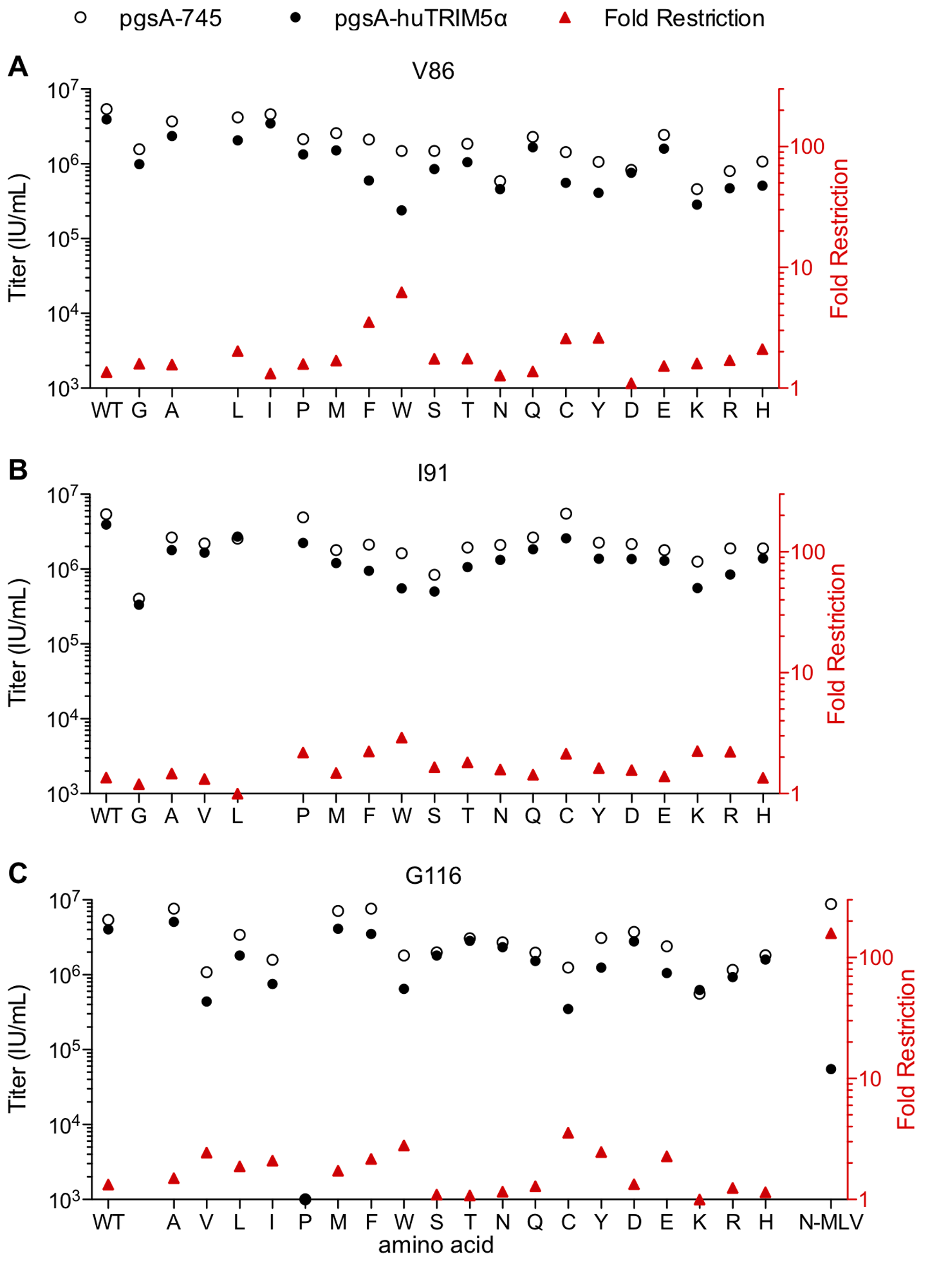

Supplement: Figure S4 — Some mutations at CA positions V86, I91 and G116 have modest effects on sensitivity to human TRIM5α. (A–C) PgsA-745 cells (open circles) or pgsA-745 cells stably expressing human TRIM5α (filled circles) were infected with VSV-G pseudotyped virions that carried a GFP-reporter gene and HIV-1NL4-3 GagPol encoding WT or mutant CA sequences. Amino acid substitutions at positions V86 (A), I91 (B), or G116 (C) are indicated on the X axis as in Figure S2. Infectious titers on each cell line (circles, referencing the left Y-axis) and the fold difference in the two titers (triangles, referencing the right Y-axis) are plotted. N-MLV was included as a positive control for restriction (C, rightmost data point). (TIF) [file ppat.1003667.s004.tif]

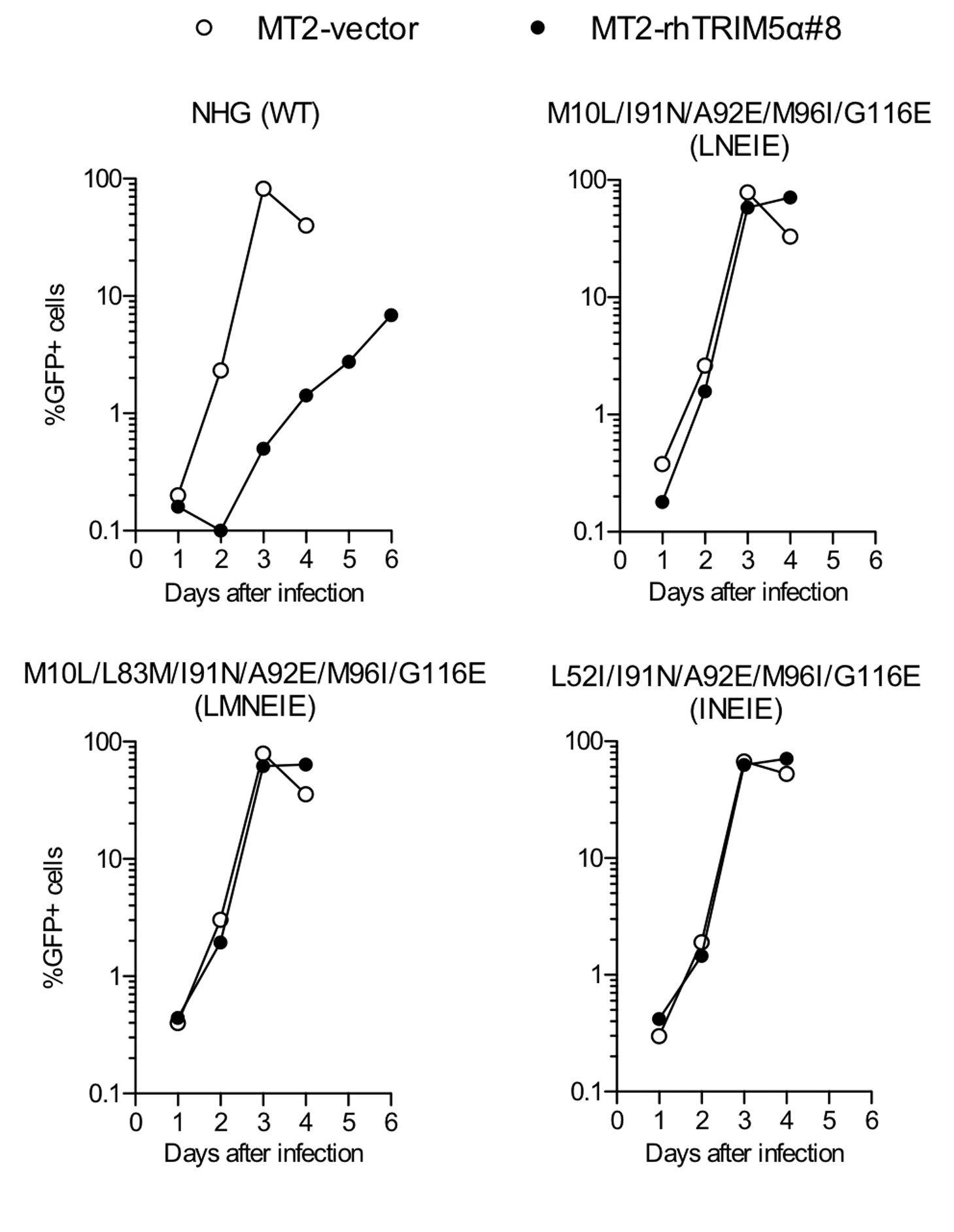

Supplement: Figure S5 — Resistance to rhTRIM5α in spreading replication assays. NHG (WT) or NHG derivatives carrying the indicated mutations were used to infect MT2-vector (open symbols) or MT2-rhTRIM5α#8 (filled symbols) cells at the same MOI. To monitor the viral spread, aliquots of cells were fixed daily for FACS analysis. The percentage of infected (GFP positive) cells is plotted against the number of days after infection. (TIF) [file ppat.1003667.s005.tif]

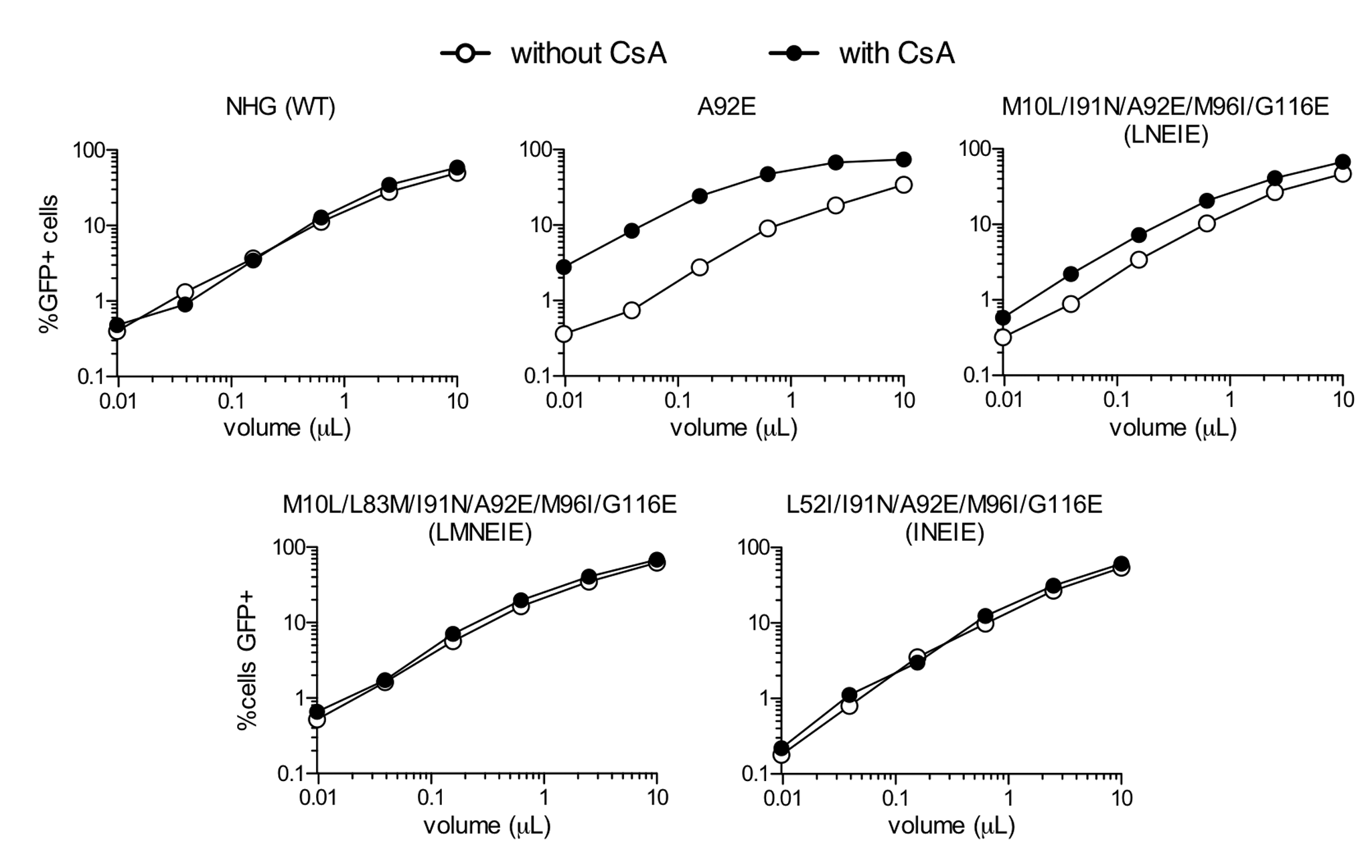

Supplement: Figure S6 — The CsA-dependent phenotype exhibited by the A92E CA mutant is not exhibited by the LNEIE, LMNEIE, or INEIE CA mutants. VSV-G pseudotyped NHG encoding WT or mutant CA sequences was used to infect HeLa cells in the absence (open symbols) or presence (filled symbols) of 5 µM CsA, as previously described [57]. The percentage of infected (GFP positive) cells is plotted versus the volume of each inoculum. (TIF) [file ppat.1003667.s006.tif]

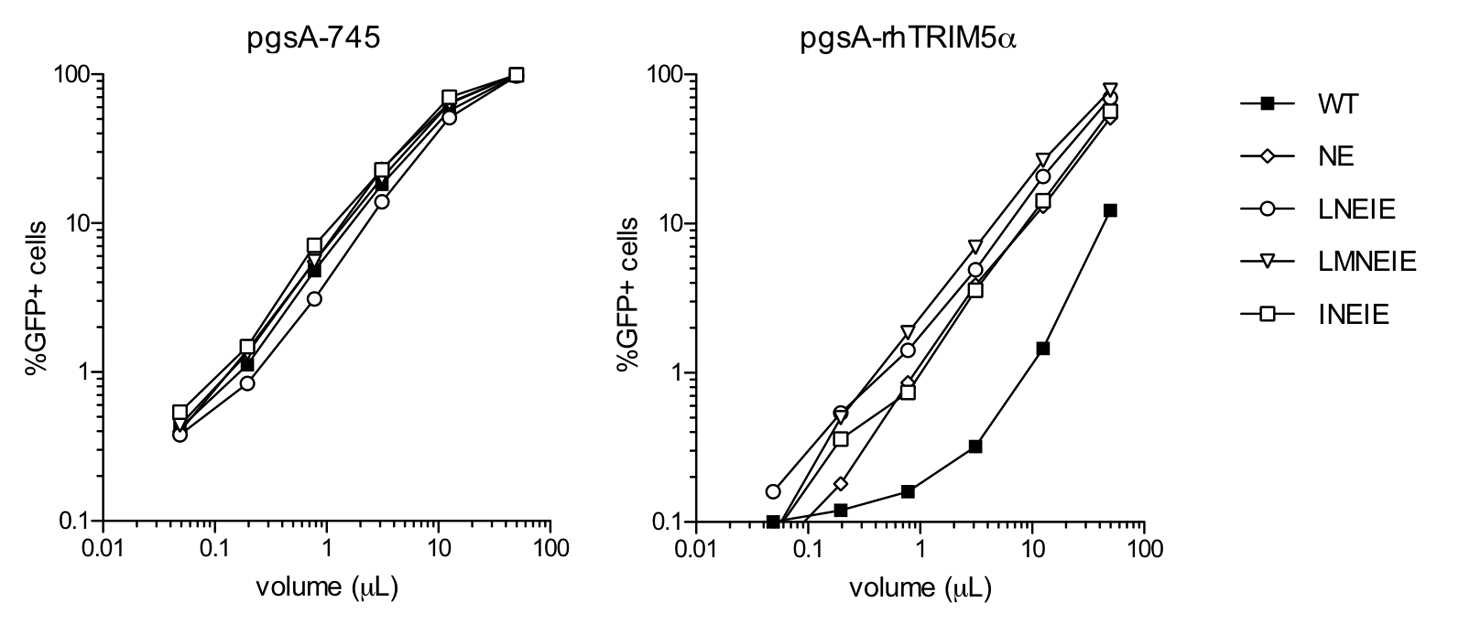

Supplement: Figure S7 — Infectivity of NE, LNEIE, LMNEIE, and INEIE CA mutants in pgsA-rhTRIM5α cells. PgsA-745 (left) or pgsA-rhTRIM5α cells (right) were infected with VSV-G pseudotyped virions that carried a GFP-reporter gene and HIV-1NL4-3 GagPol encoding WT or the indicated mutant CA sequences. The percentage of GFP positive cells is plotted for each virus dose. (TIF) [file ppat.1003667.s007.tif]

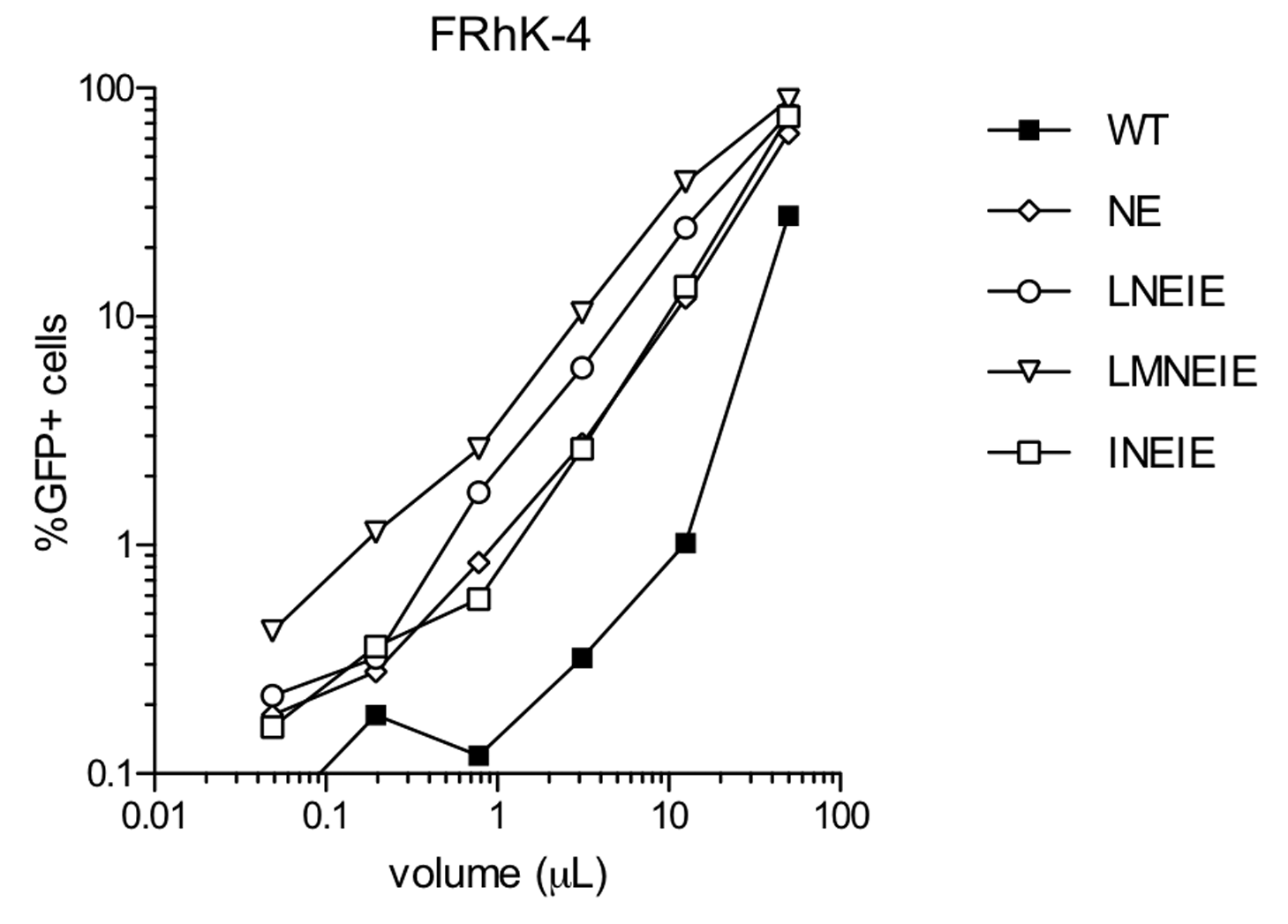

Supplement: Figure S8 — Infectivity of NE LNEIE, LMNEIE, and INEIE CA mutants in FRhK-4 cells. VSV-G pseudotyped virions that carried a GFP-reporter gene and HIV-1NL4-3 GagPol encoding WT or the indicated mutant CA sequences were used to infect FRhK-4 cells. The percentage of cells infected (GFP positive) is plotted as a function of virus dose. (TIF) [file ppat.1003667.s008.tif]

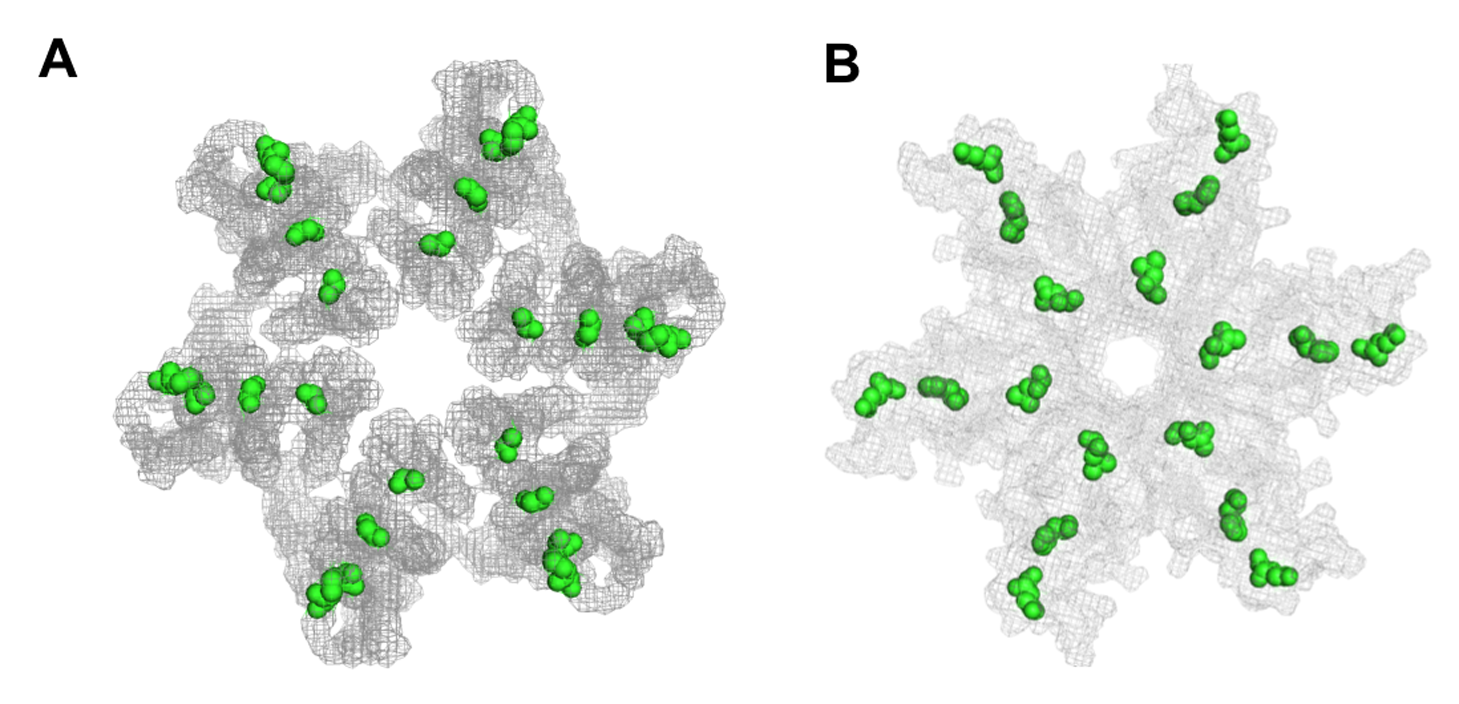

Supplement: Figure S9 — A comparison of the distribution of mutations in MLV CA and HIV-1 CA that reduce sensitivity to rhTRIM5α. (A) Positions of amino acid residues mutated in the best performing HIV-1 CA mutant (LNEIE: M10, I91, A92, M96, G116) are indicated in green on the HIV-1NL4-3 capsid hexameric structure. (B) Positions of individual amino acid residues that confer complete resistance to rhTRIM5α (L10, H114, E92) [47] are indicated on the MLV CA NTD hexameric structure. (TIF) [file ppat.1003667.s009.tif]
